# Tailored bimetallic Zn/Ni and Zn/Ag MCM-41 photocatalysts for enhanced visible-light photocatalytic tetracycline degradation

**DOI:** 10.1038/s41598-025-89522-y

**Published:** 2025-02-17

**Authors:** Mohammed Ahmed Wahba, Rabab K. Khaled, Magdah Dawy

**Affiliations:** 1 Department of Inorganic Chemistry, National Research Centre, 33 El Buhouth St. (former Eltahrir st.), Dokki, Giza, 12622 Egypt; 2 Department of Physical Chemistry, National Research Centre, 33 El Buhouth St., (former Eltahrir st.), Dokki, Giza, Egypt

**Keywords:** Bimetallic incorporation, MCM-41, Optical properties, Photocatalytic activity, Tetracycline removal, Textural features, Zn, Ni, Ag, Chemical synthesis, Process chemistry, Chemistry, Materials science, Pollution remediation

## Abstract

Novel bimetallic-doped-MCM-41(Mobil Composition of Matter No. 41) (Zn/Ni-MCM-41 (ZNM)) and (Zn/Ag-MCM-41 (ZAM)) catalysts were synthesized and characterized for their structural, textural, morphological, and optical properties. XRD analysis confirmed metal incorporation into the MCM-41 framework, while N_2_ adsorption-desorption isotherms indicated a decrease in specific surface area (1210 in pure MCM-41 to 722.86 and 700.36 m^2^/g for ZNM and ZAM, respectively) due to partial pore filling. TEM images verified this finding. Boosted absorption extending into the visible light region was detected in the metal incorporated (ZNM and ZAM) samples with additional band gaps, related to transitions in Zn^2+^, Ag^+^ and Ni^2+^ ions. Photoluminescence studies revealed efficient charge carrier separation in ZNM and ZAM. Both catalysts exhibited superior tetracycline (TC) removal from aqueous solution with efficiency (95.59% and 95.30% within one hour for ZNM and ZAM, respectively) with pronouncing visible light photocatalytic capability compared to pure MCM-41. The degradation process followed pseudo-first-order kinetics. The enhanced photocatalytic activity of ZNM and ZAM is attributed to the synergistic effects of metal incorporation, increased light absorption, and efficient charge carrier dynamics. Additionally, a possible photocatalytic mechanism for degradation of TC over ZNM and ZAM has been proposed and involvement of superoxide radicals (O_2_^•−^) and holes (h^+^) as reactive species is elucidated by radical trapping experiments. A distinct pH-dependent trend was observed in TC degradation efficiency using the ZAM photocatalyst. The efficiency gradually increased with increasing pH until reaching a maximum at pH 7, followed by a decline at higher pH values. These results demonstrate the potential of ZNM and ZAM as promising materials for removal of tetracycline antibiotic from water.

## Introduction

Freshwater, humanity’s most vital resource, is under increasing threat from a multitude of pollutants. The World Health Organization (WHO) estimates that over 47% world population lacks the use of safe drinking water, highlighting urgent actions of effective water treatment solutions^1^. Amongst various pollutants threatening the environment, tetracyclines, a prevalent class of antibiotics heavily utilized in human healthcare and animal husbandry, pose a growing environmental threat. Their widespread use leads to a significant portion, roughly 95%, being excreted unchanged and ultimately reaching aquatic ecosystems through wastewater. Unfortunately, tetracyclines exhibit high persistence in the environment, causing harm to aquatic life. Furthermore, their presence contributes to the emergence of antibiotic resistance in bacteria, jeopardizing both human health and ecological stability. This phenomenon, coupled with potential bioaccumulation through the food chain, emphasizes the urgent need for responsible antibiotic use developing effective wastewater treatment approaches to handle the release of these persistent pollutants. Traditional methods like chemical precipitation, filtration, and ion exchange, while established, often generate harmful byproducts termed toxic metabolites^2–5^. These byproducts can pose significant health risks, necessitating the exploration of more sustainable approaches. Among these approaches, advanced Oxidation Processes (AOPs) offer a proficient avenue for water treatment.

AOPs photocatalysis utilizing semiconductors processes have emerged as effective methods for controlling organic pollutants in water^6,7^. AOPs address a key limitation of traditional methods by minimizing the generation of harmful byproducts^8^. Significant research efforts have focused on developing efficient photocatalysts^9–14^. However, two key challenges hinder their widespread application. (i) limited light absorption as most photocatalysts, like TiO_2_ and ZnO, primarily UV light, which establishes only 3–5% of the solar radiation^15–17^ (ii) rapid charge-carriers recombination^18^. To address these limitations, several exertions were directed for developing visible-light-driven photocatalysts and extending the reactive species life span. One approach involves engineering photocatalysts with different morphologies and sizes to optimize light absorption and charge separation^14,19^. While reducing particle size enhances a photocatalyst’s surface area, it also creates an agglomeration problem. Furthermore, separating of these tiny particles after use becomes challenging. To address these issues, researchers are exploring strategies to assemble these nanoparticles into larger microstructures or incorporate them within various support materials. These support materials, like zeolites or silica, can help prevent nanoparticle clumping, separation of generated hole-electron charges, and simplify their recovery from the reaction system after use^20^.

MCM-41, mesoporous silica, possesses boasts a superior pore volume, a high surface area, tunable chemical composition, as well as a functionalizable surface. However, it exhibits intrinsic photocatalytic inactivity^21^. This limitation arises from the inherent light insensitivity of its constituent elements, silicon and oxygen. Enhancing the catalytic performance of MCM-41 could be attained through functional group incorporation. The large pores and extensive surface area of MCM-41 facilitate inclusion of various guest components, including metals, metal oxides, and metal complexes. This strategic incorporation significantly enhances the material’s catalytic activity. A diverse range of approaches exist for modifying mesoporous materials like MCM-41. Some key strategies including: replacing silicon within MCM-41 structure with metal ions that generates acidic or redox-active sites, enabling the material to catalyze a wider range of reactions. The framework of MCM-41 allows for the creation of active sites through various pre- and post-synthesis techniques. This approach offers precise control over the material’s properties and catalytic activity. The majority mesoporous silica modification methods leverage the abundant Si-O-H groups present on the material’s surface. These silanol groups serve as anchor points for the attachment of M ions or coupling agents, facilitating further functionalization^22–24^.

Significant research efforts have focused on incorporating various elements into the MCM-41 framework, including: Al, Ga, In, Zn, Cd, Ti, V, Fe, Cu, Nb, Mo, Zr, La, Ce^22–27^. Metal ions inclusion into MCM-41 offers a broader spectrum of functionalities due to the unique properties imparted by d-electron confinement within their nanoscale pores^28^. These modifications aim to create stable and effective materials for like adsorption, photodegradation, separation techniques, and ion exchange chromatography applications. This study builds upon this exciting field by exploring the potential for metal-modified mesoporous silica materials with boosted adsorption and solar photocatalytic activity. Pristine as well as bimetallic incorporated MCM-41 using combinations of Zn^2+^ with Ag^+^/Ni^2+^ were synthesized via a surfactant-assisted precipitation process. The influence of M-ions incorporation on structural, morphological, textural, and optical characteristics of MCM-41 framework was then systematically investigated. The combination of Zn with Ni or Ag is expected to exhibit synergistic effects in enhancing photocatalytic activity. ZnO is known for its photocatalytic properties. Ni and Ag can act as co-catalysts, potentially improving charge separation, inhibiting electron-hole recombination, and enhancing light absorption. It is assumed that incorporating these metals will modify physicochemical traits of MCM-41, leading to: creation of diverse active sites; enhanced light absorption capabilities; potentially increased ion-exchange capacity. The materials’ efficacy for removing of tetracycline, a common antibiotic, from solution was evaluated. In particular, we are interested in the potential synergistic effect between ZnO and Ag^+^ or Ni^2+^ within the mesoporous framework. To gain further insights into the degradation mechanism, radical trapping experiments were conducted to identify the primary reactive oxygen species (ROS) involved. Furthermore, the influence of solution pH on TC degradation efficiency using the ZAM sample was systematically evaluated. This synergistic effect could lead to a broadened spectrum of activity and enhanced removal efficiency for tetracycline under visible light.

## Experimental

### Materials and experimental methods

Hexadecyltrimethylammonium bromide (CTAB, MW 364.45) was purchased from Acros Organic (Belgium), sodium silicate (Na_2_SiO_3_, MW 122.06), zinc nitrate hexahydrate (extra pure, Zn(NO_3_)_2_·6H_2_O, MW 297.48) from Alphachemika (India), nickel nitrate hexahydrate (purified LR, Ni(NO_3_)_2·_6H_2_O, MW 290.80), silver nitrate (AgNO_3_, MW 169.87) from Ld.Fine-Chemlid, and sodium hydroxide pellets (MW 40 g/mol) from Sigma-Aldrich. The different scavengers: disodium ethylenediaminetetraacetate (EDTA-2Na), IPA (isopropanol), ASA (ascorbic acid) and TCM (tetrachloro methane) were purchased from Alfa-Aesar.

### Preparation of pure MCM‑41 support and bimetallic (Zn/Ni) and (Zn/Ag) modified MCM‑41

MCM-41 was synthesized through a surfactant-assisted precipitation process. CTAB (3.64 g) was dissolved in NaOH solution at 55 °C to form a homogeneous mixture. Subsequently, sodium silicate solution (10.158 g Na_2_SiO_3_ in 80 mL distilled water) was added dropwise, and the pH was adjusted to 10. The reaction mixture was stirred at room temperature for 24 h, followed by filtration, washing, and drying at 70 °C. The final product was calcined at 550 °C for 6 h to obtain pure MCM-41, denoted as pure M. To prepare Zn/Ni-modified MCM-41 (ZNM) and Zn/Ag-modified MCM-41 (ZAM), uncalcined CTAB-MCM-41 (3.0 g) was dispersed in water. Aqueous solutions containing the desired amounts of zinc nitrate, nickel nitrate, or silver nitrate (to achieve a Zn/Si and Ni/Si or Ag/Si atomic ratio of 4%) were added to the MCM-41 dispersion. The mixture was stirred at room temperature for 1 h, then at 80 °C for 5 h. The resulting solid was dried at 70 °C and calcined at 550 °C for 6 h to remove organic templates.

### Removal of tetracycline

The photocatalytic performance of the synthesized pure M, ZNM, and ZAM materials was assessed by their ability to degrade tetracycline (TC) under direct solar light irradiation. In a typical experiment, 0.05 g of catalyst was added to 50 mL of a 40 mg/L TC solution. The mixture was kept in the dark for 30 min to establish adsorption-desorption equilibrium before exposure to sunlight for 1 h. At specific time intervals (2, 5, 10, 15, 20, 25, 30, 40, 50, and 60 min), 3 mL of the irradiated mixtures were collected. Subsequently, the samples were centrifuged for 5 min to separate the powder from the solution. The concentration of TC in the solution was determined by measuring the absorbance at a specific wavelength using a Cary 100 spectrophotometer^29^. All test solution batches were carried out during the month of July, subjected to continuous stirring under natural sunlight exposure between 12:00 p.m. and 3:00 p.m. at the NRC Institute in Cairo, located at a latitude of 30° 3′ 45.47″ N and a longitude of 31° 14′ 58.81″ E. During this period, fluctuations in sunlight intensity were minimal, with the average intensity ranging from 680 to 700 W/m^2^, as determined using a solar power meter. The active species capture assay was performed to find out what reactive species were produced during photocatalysis through dissolving various scavengers such as EDTA-2Na (used to capture h^+^ (holes)), IPA (used to confine hydroxyl radicals (^•^OH)), ASA (used to trap electrons (e^−^)) and TCM (superoxides (^•^O_2_) radicals scavenger) into the aqueous mixture of TC^30,31^. Before the addition of the photocatalyst, scavengers were mixed separately into the TC solution where 1 mM of every scavenger employed in the photocatalysis reaction^32,33^. All experiments were conducted in triplicate, with consistent results across three independent trials. The effect of pH value on the TC degradation was studied using 50 mL of 40 mg L^− 1^ of TC solution on ZAM sample for 60 min, whereas the pH values varied from 3 to 9 and the pH was adjusted with 0.12 M HCL and 0.12 M NaOH for acidic and alkaline media, respectively.

### Characterization of materials

XRD patterns were collected using a PANalytical X’Pert PRO diffractometer to determine the crystalline phases of the samples. N_2_ adsorption-desorption isotherms were measured at liquid nitrogen temperature (-196 °C) using a NOVA 3200 instrument to calculate specific surface area (S_BET_), pore size distribution (using DFT method), and pore volume. Prior to analysis, samples were degassed under vacuum (10^− 4^ Torr) at 300 °C overnight. The morphology and elemental composition of the samples were examined by SEM coupled with EDS unit using a Quanta 250 FEG microscope while TEM micrographs were obtained using a JEOL JEM-2100 microscope. The functional groups present in the samples were identified through FTIR using a Brucker Vertex 80 V spectrometer in the range of 4000 –400 cm^−1^. Photoluminescence (PL) spectra were measured on a Jasco FP-6500 spectrofluorometer (made in japan) using Xenon arc lamp 150 W to assess the charge carrier recombination behavior. The optical properties of the samples were investigated using UV-Vis diffuse reflectance spectroscopy on a JASCO V-570 spectrophotometer. The band gap energy was determined from the DRS data using the Kubelka-Munk function and Tauc plot analysis, assuming both direct and indirect transitions (*n* = 0.5 and 2, respectively). The Kubelka-Munk function (F(R)) is related to the absorption coefficient (α) and scattering coefficient (s) by the equation F(R) = (1-R)^2^/(2R) = α/s, where R is the reflectance. The band gap energy (E_g_) was calculated from the Tauc plot, which is a plot of (αhν)ⁿ versus photon energy (hν), where α is the absorption coefficient, h is Planck’s constant, ν is the light frequency, and n is the exponent associated with the type of electronic transition.

### Data analysis

The study evaluated the TC removal onto pure M, ZNM and ZAM samples by using various models to determine the photodegradation efficiency, kinetic mechanism, and possible TC degradation mechanism. Based on the experimental data, the photodegradation efficiency (PE) was calculated using the following equation:

$${\text{PE}} = {\text{A}}_{{\text{t}}} /{\text{A}}_{0} = {\text{C}}_{{\text{t}}} /{\text{C}}_{0}$$

where A_0_ and C_0_ are the initial absorbance and concentration of TC, respectively, and A_t_ and C_t_ are the absorbance and concentration of TC after a certain irradiation time^29^.

The kinetic behavior of the pure M, ZNM and ZAM based photocatalytic system for TC degradation was assessed considering the linear fitting of the degradation results with the theoretical assumptions of both pseudo first-order and pseudo second-order models and their representative equations are^29^:

$${\text{Ln}}\left( {{\text{C}}_{{\text{t}}} /{\text{C}}_{0} } \right) = - {\text{k}}_{{\text{1}}} {\text{t}}\quad {\text{Pseudo}}\;{\text{first - order}}$$

$${\text{1}}/{\text{C}}_{{\text{t}}} = {\text{1}}/{\text{C}}_{0} - {\text{k}}_{{\text{2}}} {\text{t}}\quad {\text{Pseudo}}\;{\text{second - order}}$$

where C_t_ is the TC concentration at time t; C_0_ is the initial TC concentration.

## Results and discussion

### XRD analysis

**Fig. 1:**
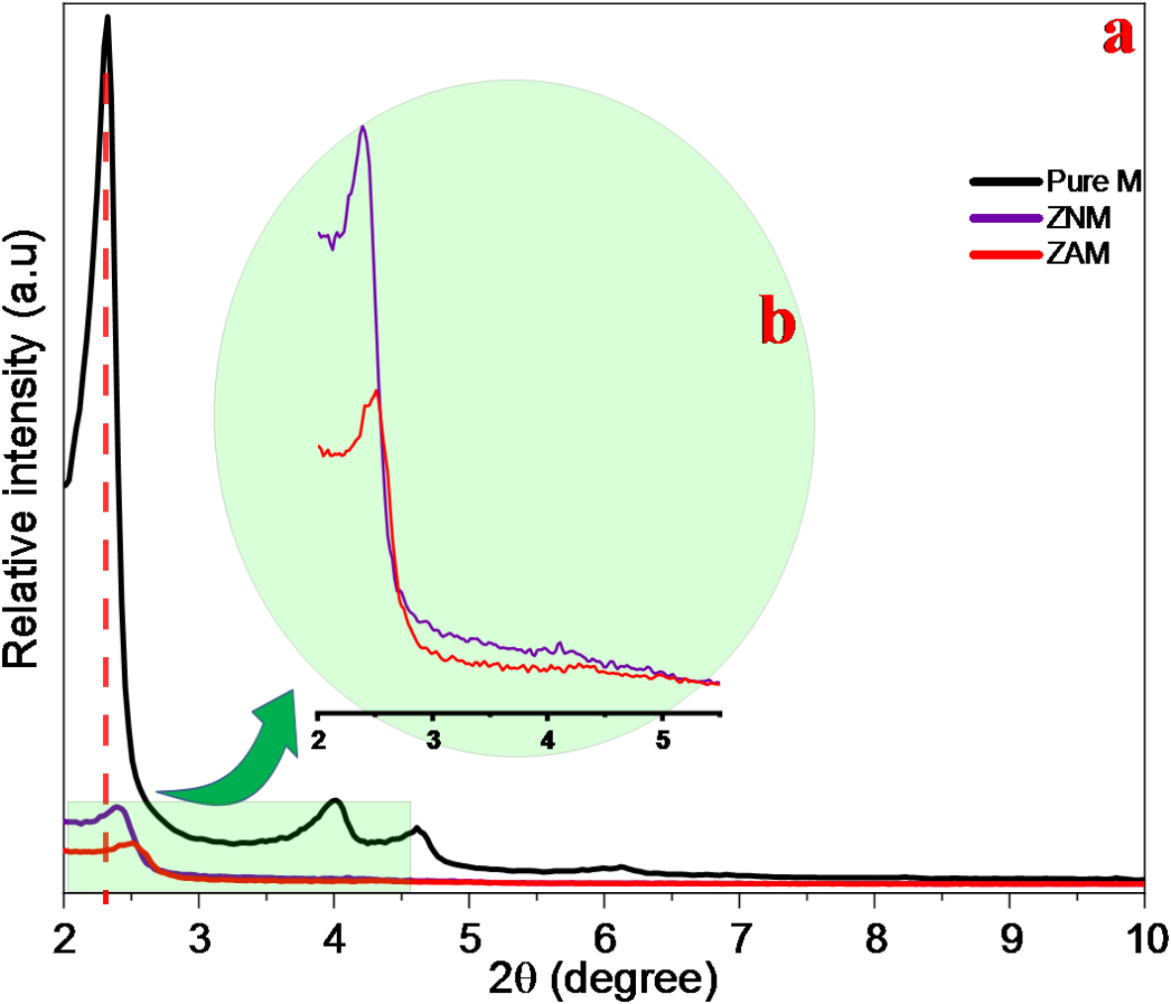
Low-angle XRD patterns of pure M, ZNM, and ZAM samples. (**a**) Full pattern and (**b**) Magnified pattern highlighting lower intensity peaks.

The low angle (2–10 degrees) XRD of the pure support M, ZNM, and ZAM bimetallic incorporated samples are shown in Fig. 1a. 4-discrete diffraction peaks were observed for the pure M refering to distinct MCM-41 structure. Clearly, the three diffraction 2.34°, 4.03°, and 4.7° ascribed to the (100), (110), and (200) hexagonal unit cell planes, signifying establishing of well-organized hexagonal pores^34^. An additional peak at 6.2° is shown connected to the 300 plane that is ocasionally appear in exceptionally highly-ordered MCM-41 structures.

Through detalied XRD patterns examination, (Fig. 1b) prominent changes emerge upon comparison the patterns of pure M and ZAM and ZNM incorporated compostions. All peaks become less sharp and is subjected to a noticeable broadening. The intensity of the main peak (100) is markedly influenced. It becomes weaker in the ZAM and ZNM samples, and this decline is more pronounced in ZAM than ZNM. Notably, the ZAM sample completely lacks the additional peaks typically observed for MCM-41, labeled (110), (200), and (300). On the other hand, the ZNM sample retains the (200) peak, but it became weaker. Additionally, both ZAM and ZNM show slight shifts (higher 2θ values) in comparsion to the pure M sample. The broader peaks, the absence of certain peaks, and the lower intensity of all peaks in ZAM and ZNM samples as well as 2θ shifts compared to pure MCM-41 act as strong indicators that the metals have been incorporated and integrating the silica matrix^35,36^. These XRD variations reflect modifications in the mesoporous structure.

Although Zn/Ag and Zn/Ni ions incorporation partailly disrupts the mesoporous channels-ordering. Interestingly, the hexagonal structure seems to be somewhat preserved. A significant observation is the (100) diffraction line shift to higher angles (2θ). This observed shift might suggest that incorporated metal species strongly interact with MCM-41 framework^37^. However, the magnitude of this shift is greater for ZAM (0.17°) compared to ZNM (0.05°). This implies more unit cell contraction and a reduction in the average distance between pore centers in the ZAM sample.

XRD patterns was utilized to estimate the difference in pore size between pure M-MCM-41 samples. The analysis focused on the (100) peak and used Bragg’s Law $\:{a}_{0}=\:2{d}_{100}/\sqrt{3\:}$. to calculate the spacing between pores-centers, also known as the unit cell parameter ($\:{a}_{0}$). The d-space of the (100) diffraction is 37.71 Å for the pure M sample, which is assigined to $\:{a}_{0}$ of 43.54 Å. However, the XRD results for ZNM and ZAM samples indicated a shift to lower d spacing, with the (100) diffraction d-spacing decreasing to 36.92 and 35.16 Å, respectively, corresponding to pore repeat distances $\:{a}_{0}$ of 42.63, and 40.60 Å, respectively. These results suggest a slight shrinkage in the unit cell of ZNM and ZAM when contrasted with the pure M. The pore shrinkage is likely related to the (Si) atoms replacement by metal atoms (M) in the framework, causing it to contract. The reasons for the variation in the unit cell parameter ($\:{a}_{0}$) might be related to the inherent disorder in the atomic arrangement of MCM-41. Metal ions inclusion might influence the degree of polymerization of silicate molecules within the framework. Compared to pure silicate, Zn/Ni-silicate and Zn/Ag-silicate might condense more readily, leading to slightly different unit cell parameters.

The mesopore diameters (W_d_) of the pure M, ZAM and ZNM were determined using a well-established equation incorporating the unit cell parameter ($\:{a}_{0}$) obtained from the XRD analysis (Eq.)^22,38,39^ $\:{W}_{d}=cd\sqrt{(\rho\:\:{V}_{p}/(1+}\rho\:\:{V}_{p})$). This equation accounts for (c) as a constant value, the (d) as the (100) d-distance, (ρ) pore-wall density, and (V_p_) the pore-volume. The W_d_ values for pure M, ZAM and ZNM samples (Table 1) revealed a small reduction (0.075 nm) in ZNM in related to the pristine material. However, a more pronounced decline of 0.255 nm was observed in ZAM. The observed differences in both ($\:{a}_{0}$) and ($\:{W}_{d}$) provide compelling evidence for the successful incorporation of Zn/Ag and Zn/Ni metals into the framework of the MCM-41 samples. The existence of these metal species likely influences mesopores structural and dimensional properties.

### N_2_ adsorption-desorption isotherms

**Fig. 2:**
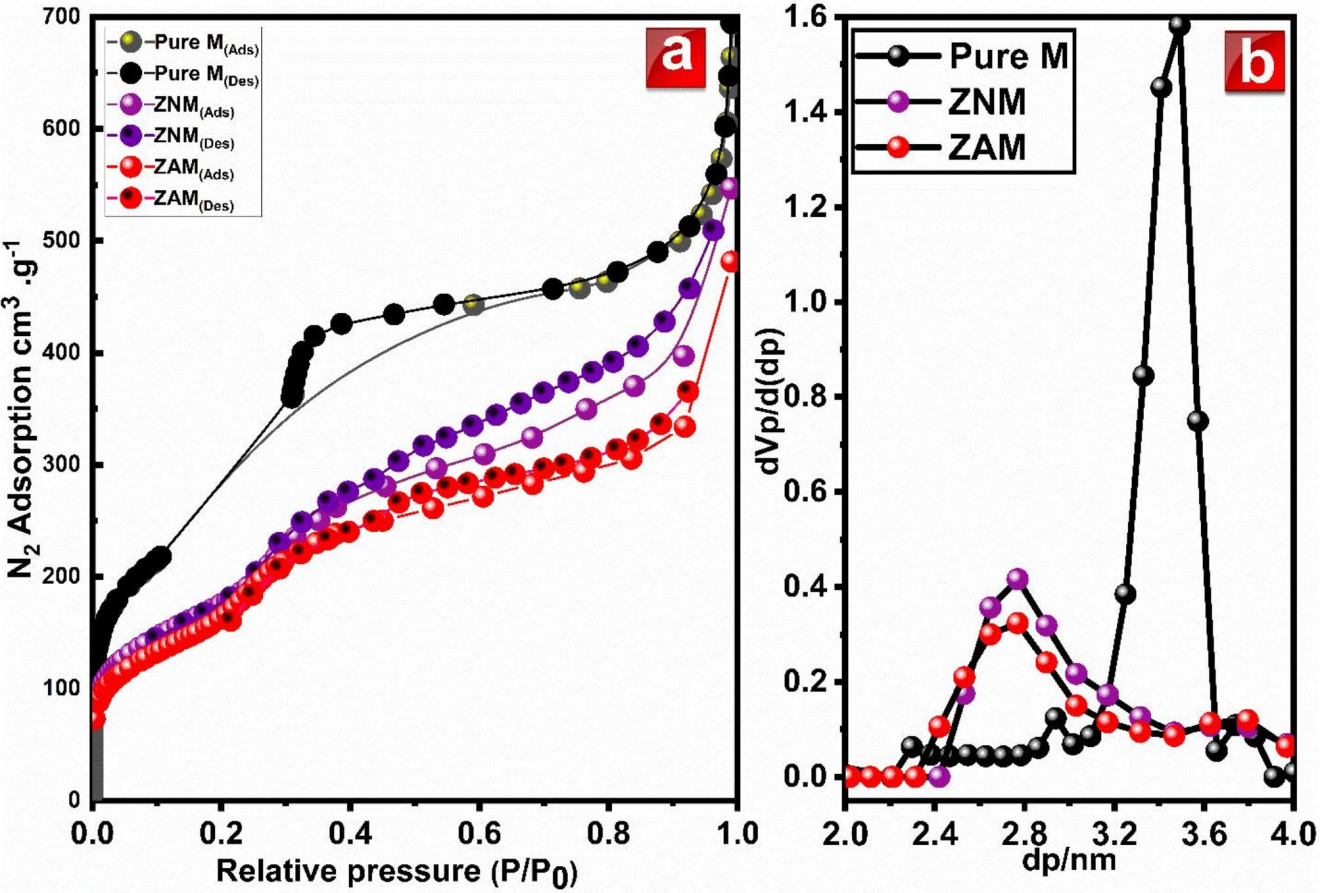
(**a**) N_2_ adsorption–desorption isotherms and (**b**) pore size distribution of pure MCM-41, ZNM and ZAM samples.

Figure 2a reveals the N_2_ adsorption-desorption behavior for all synthesized materials at different pressures (P/P_0_: 0.005–1.00). Notably, all samples demonstrated type IV isotherms, signifying a three-stage process. For MCM-41, (i) monolayer-multilayer adsorption (0.005 < P/P_0_ < 0.1): at low pressures, the inclined isotherms suggest an exsitance of micropores. These tiny pores strongly attract nitrogen molecules, resulting in a single, tightly-packed layer on their surface^40^. (ii) Capillary condensation (0.31 < P/P_0_ < 0.59): this range in the pure MCM-41 support exhibits a distinct hysteresis loop. This loop signifies capillary condensation, where nitrogen molecules fill and condense within the highly organized, symmetrical mesopores of MCM-41. The steep slope of the loop reflects the high regularity of these pores, further validated by XRD data. (iii) multilayer adsorption on the outer surface (0.59 < P/P_0_ < 0.8): Shows a nearly horizontal region, indicating limited further adsorption. However, at high P/P_0_ (0.8-1.0), the N_2_ adsorption rise sharply, revealing dominance gas adsorption on larger pores. This significantly boosts the overall gas adsorption capacity of the material. Notably, the absence of a distinct plateau near P/P_0_ = 1 suggests an unsaturated adsorption phenomenon at high pressures. The pure M exhibited a remarkable S_BET_ of 1210 m^2^/g.

Compared to their parent MCM-41, Zn/Ni and Zn/Ag-incorporated MCM-41 samples display distinct differences in N_2_ adsorption, especially in the multilayer zone. These variations arise from altered surface properties. Both samples exhibits a lower maximum adsorbed volume (consistent with its lower surface area). Furthermore, the capillary condensation step in the range 0.18–0.99 for ZNM, 0.43–0.97 for ZAM shows a less pronounced slope, suggesting a broader pore size distribution upon introducing of Zn/Ni and Zn/Ag in the MCM-41 framework. Notably, adsorption increases at higher P/P_0_ owing to multilayer formation on the external surface. The distinct H_3_ hysteresis loop, persisting even at high pressures, reinforces the influence of the pore network and points towards slit-like mesopores^41,42^.

Table 1 summarized the textural characteristics of the pure M, ZAM and ZNM samples estimated from the adsorption–desorption isotherms. In contrast to the pure M material’s with S_BET_ of 1210 m^2^/g, ZNM and ZAM, recorded 722.86, 700.36, respectively. This decline in S_BET_ area stems from partial inclusion of metal ions within the pores as well as metal centers deposition on surface of MCM-41, as evidenced by TEM micrograph^43^.

Interestingly, metals species impact this “pore plugging” effect differently. ZAM showed more pronounced decline in S_BET_ referring to further silver migration within the MCM-41 framework. This migration could further constrict accessible mesopores, amplifying the surface area reduction^41,42^.

Figure 2b displays the plots of DFT pore size distribution (PSD) of pure M, ZAM and ZNM. The pure sample exhibits a confined mesopore size distribution, while discernible diversity is observed in the pore-size distributions in ZAM and ZNM samples. The reduced consistency in PSD in ZAM and ZNM samples implies presence of varying degrees of disorder in these materials, which may be linked to an asymmetrical pore-arrangement or a heightened defects concentration in the pore framework. This suggests a potential source of variability and asymmetry in the pore structure of ZAM and ZNM in contrast to the more uniform structure of pure M. the ZNM and ZAM samples recorded a smaller average pore diameters (**D**_**p**_) of (2.77 nm) than those of pure MCM-41. Moreover, a similar trend was noted in the mesoporous volume (V_p_), which exhibited a decrease in the ZAM and ZNM samples, consistent with the observed reduction in pore diameter. The absence of a consistent rule governing this variation can be ascribed to the amorphous nature of these materials, wherein bond angle and length undergo dynamic changes, resulting in unpredictable modifications to the pore characteristics.

The calculation of wall thickness followed the formula $W_{t}= (2/√3) * d_{100}-- D_{p (DFT)}$, indicating a shift from 0.844 nm in the pure M to 1.49 and 1.29 nm for ZNM and ZAM respectively. This alteration aligns with findings from XRD analysis. The observed increase in wall thickness in these samples, relative to pure MCM-41, implies a structural modification resulting from the inclusion of Zn/Ag and Zn/Ni ion species into the mesopores structure. Comparable results have been documented in studies involving the integration of Ti, V, and Mo into MCM-41, demonstrating an augmentation in wall thickness^44–47^. The significance of these changes in wall thickness lies in the metal ions implications on the inner pore-framework. It is probable that these modifications influence the ordering of silica atoms inside the pore, resulting in a denser or thicker wall compared to the pure M. The observed thicker walls in the ZNM and ZAM samples may be linked with the enhancement in the extent of zinc, nickel and silver silicates polymerization within the pore wall. This enhancement could contribute to a more robust structural stability, representing a reliable improvement in the overall stability of the material.

### Morphological analysis

#### SEM and EDX

**Fig. 3:**
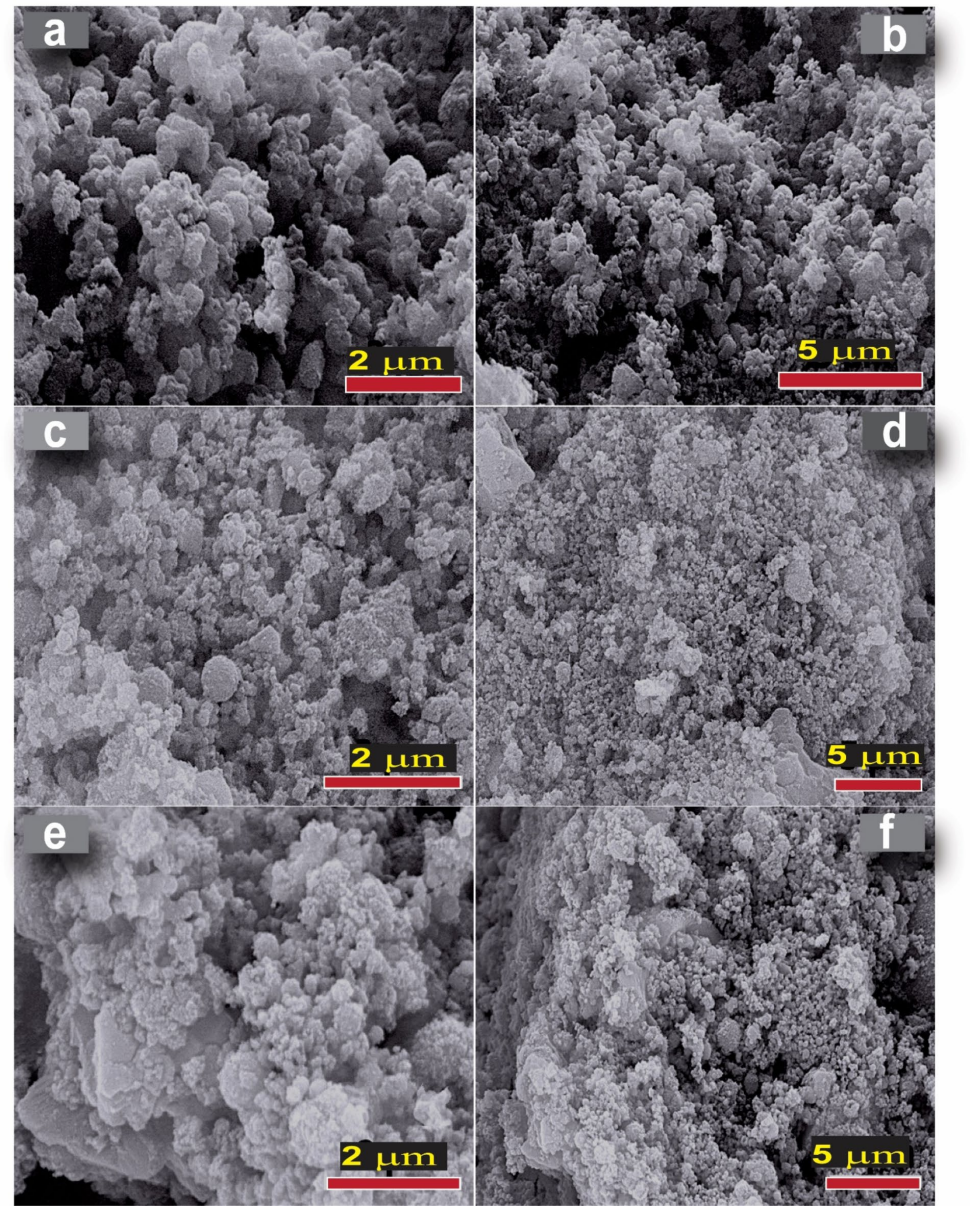
SEM images at low and high magnifications, respectively, of Pure M (**a**, **b**), ZNM (**c**, **d**) and ZAM (**e**, **f**) samples.

**Fig. 4:**
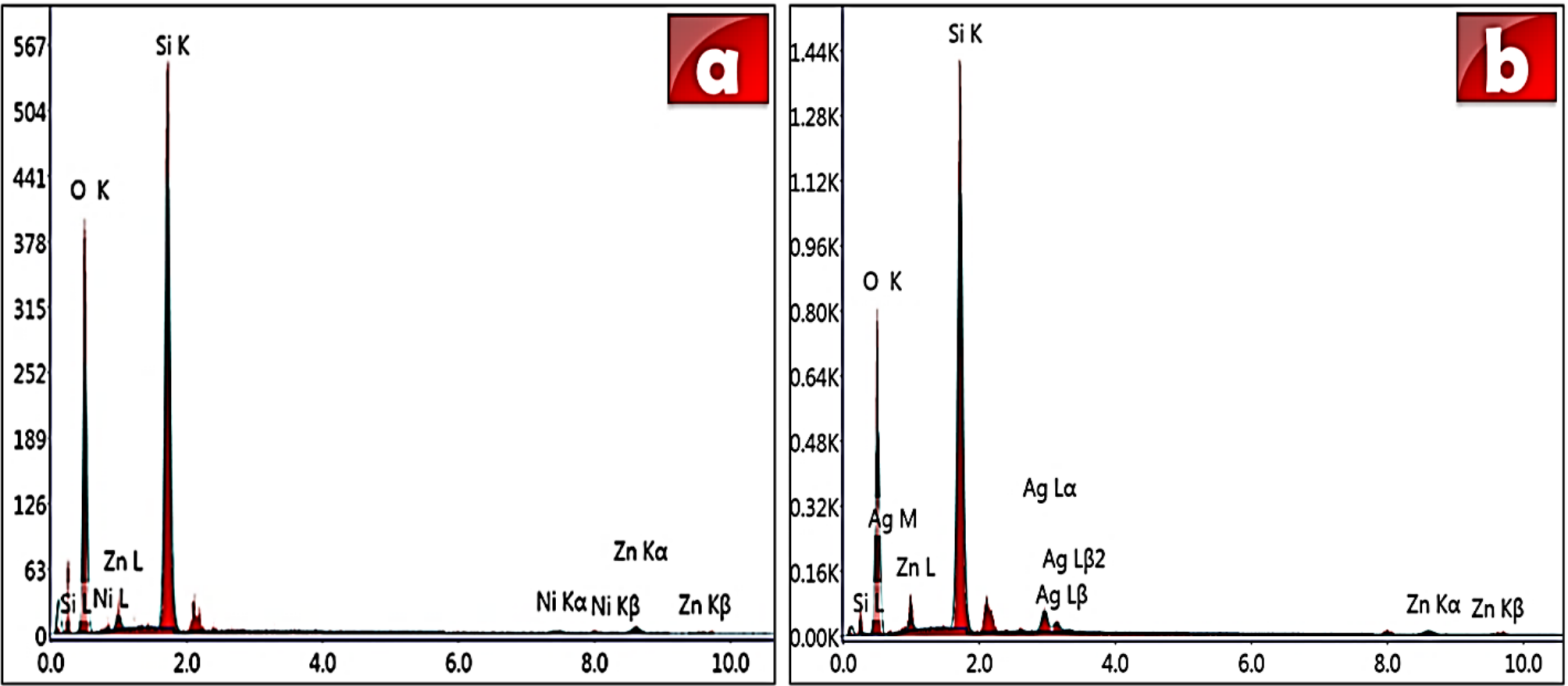
Energy dispersive spectroscopy (EDX) analysis of (**a**) ZNM and (**b**) ZAM samples.

Figure 3 presents SEM micrographs of the pure M, ZAM and ZNM compositions. The pure M (Fig. 3a and b) reveals distinct morphologies with nearly spherical particles. In contrast, ZNM and ZAM (Fig. 3c–f) exhibit an altered morphology with a slightly larger spheres, spongy pattern alongside the appearance of sheet-like particle. This suggests a complex interaction between metal ions and the MCM-41 framework, influencing growth patterns and potentially leading to structural variation. These observations demonstrate the successful incorporation of bimetallic ions (Zn & Ni, Zn & Ag) into the MCM-41 framework, highlighting the versatility in achieving morphological diverse and structural adjustments through compositional control. EDX analysis was employed to evaluate the purity and elemental composition of the synthesized nanopowders. The EDX spectra confirmed the presence of Si and O as the primary constituents, along with Zn, Ni, and Ag as dopant elements (Fig. 4). The absence of any additional elements indicates the high purity of the prepared samples.

#### TEM analysis

**Fig. 5:**
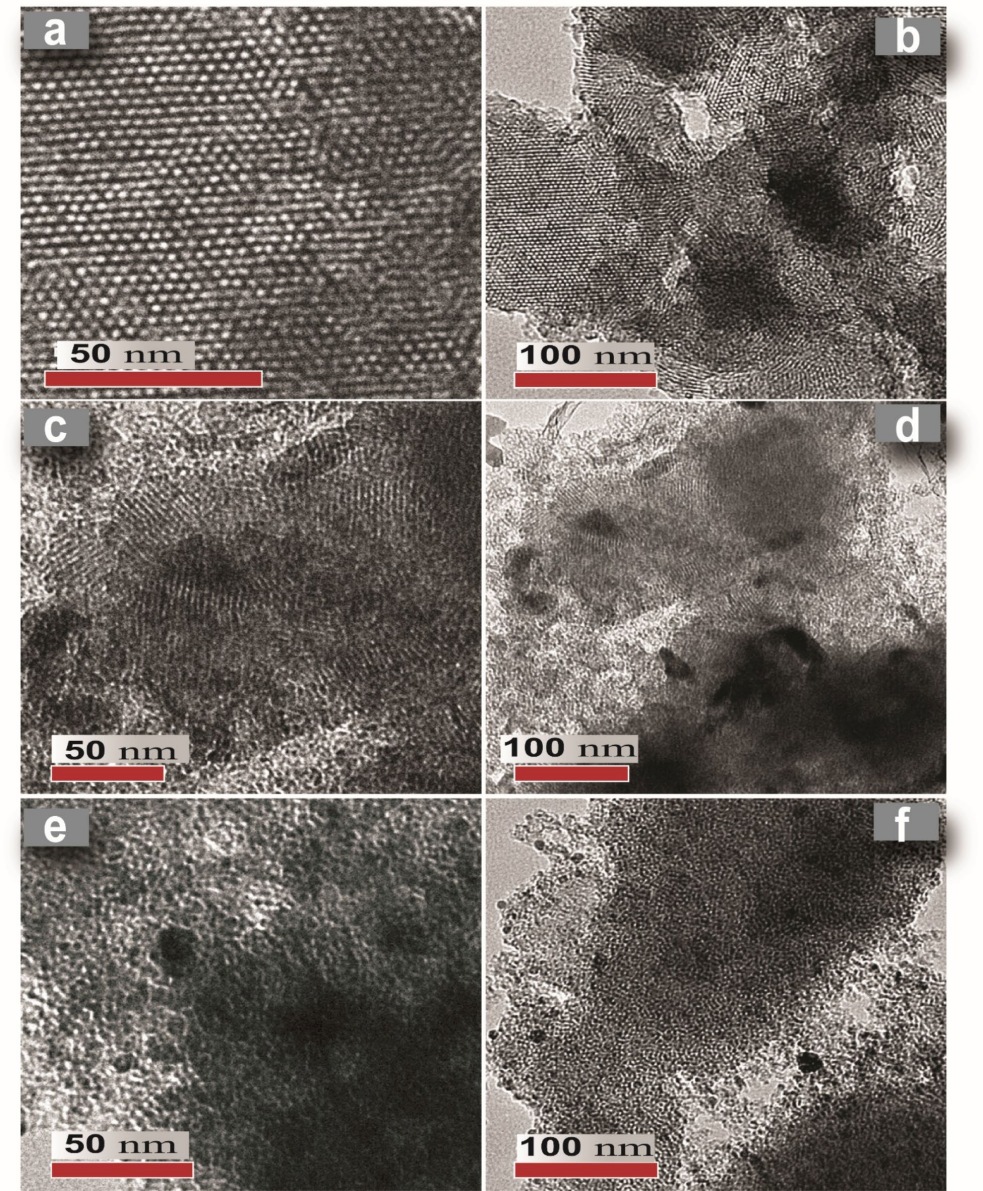
High-resolution transmission electron microscopy (HRTEM) images revealing honeycomb pattern at high and low magnification for (**a**, **b**) pure M, (**c**, **d**) ZNM, and (**e**, **f**) ZAM samples.

**Fig. 6:**
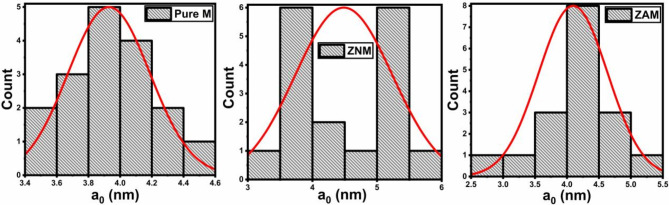
Histogram illustrating the average cell parameter (a_0_) for pure M, ZNM, and ZAM samples.

Figure 5 presents TEM micrograph of pure M, ZAM and ZNM samples, providing insights into their structural investigation. The TEM image of pure M (Fig. 5a) reveals a uniform linear pattern aligned vertically along the pore channels, indicative of a highly ordered arrangement. This observation aligns with XRD findings. Figure 5b displays a distinct hexagonal pore pattern, where each pore is encircled by 6-adjacent pores. The single-phase domain, consistent mesoporosity, and thin sample thickness suggest a well-structured MCM-41 sample. The measured pore centers distance, representing the cell parameter (a_o_), is 3.93 nm (Table 1). The D_p_ estimated from the TEM micrograph is 2.4 nm, consistent with the typical values reported for calcined MCM-41. TEM images of metal modified samples (Fig. 5c–f) disclose a pore structure similar to pure M sample, although with some irregularities and defects probably caused by metal ion inclusion. The presence of denser metal ions (Ni, Zn, and Ag) compared to silicon in the MCM-41 matrix results in the appearance of darker spots within the TEM images, as reported in previous studies^43,48^. Estimated particle sizes for Zn, Ni and Ag are 25.7 nm and 5.96 nm, respectively (Fig. 5). However, the comparatively bigger size of these metal centers in comparison to the calculated pore diameters suggests their location outside the MCM-41 pores rather than within them. TEM image analysis (Fig. 6) revealed average cell parameters of 4.486 nm and 4.097 nm for ZNM and ZAM samples, respectively, as determined by measuring the distance between adjacent parallel lines. These values are less the corresponding XRD-derived cell parameters by 0.091and 0.29 nm (Table 1). Additionally, calculated average pore diameters from TEM images were 2.3 nm and 2.37 nm for ZNM and ZAM, respectively, smaller than the pure M pore width. These findings align with the pore size variations observed between the pristine and metal-modified MCM-41 materials deuced from XRD data.

**Table 1:** Structural and textural properties of the pure M, ZNM and ZAM incorporated samples.

Samples	XRD			*N*_2_ adsorption-desorption isotherms				TEM
	d_100_ (nm)	a_0_ (nm)	W_d_ (nm)	S_BET_ (m^2^.g^− 1^)	V_*p*_ (cm^3^/g) DFT	D_*p*_ (nm) DFT	W_t_ (nm)	a_0_ (nm)
Pure M	3.771	4.35	3.735	1210	1.30	3.51	0.844	3.93
ZNM	3.692	4.26	3.66	722.86	0.74	2.77	1.49	4.486
ZAM	3.516	4.06	3.48	700.36	0.52	2.77	1.29	4.097

*(a_0_) average lattice parameter: $a_{0} = 2d_{{100}} /\surd 3$; V_p_: pore volume; D_p_: average pore size, calculated by DFT model; Wall thickness $W_{t} = \left( {2/\surd 3} \right)*d_{100}{-}D_{p(DFT)}$.

### Optical analysis

**Fig. 7:**
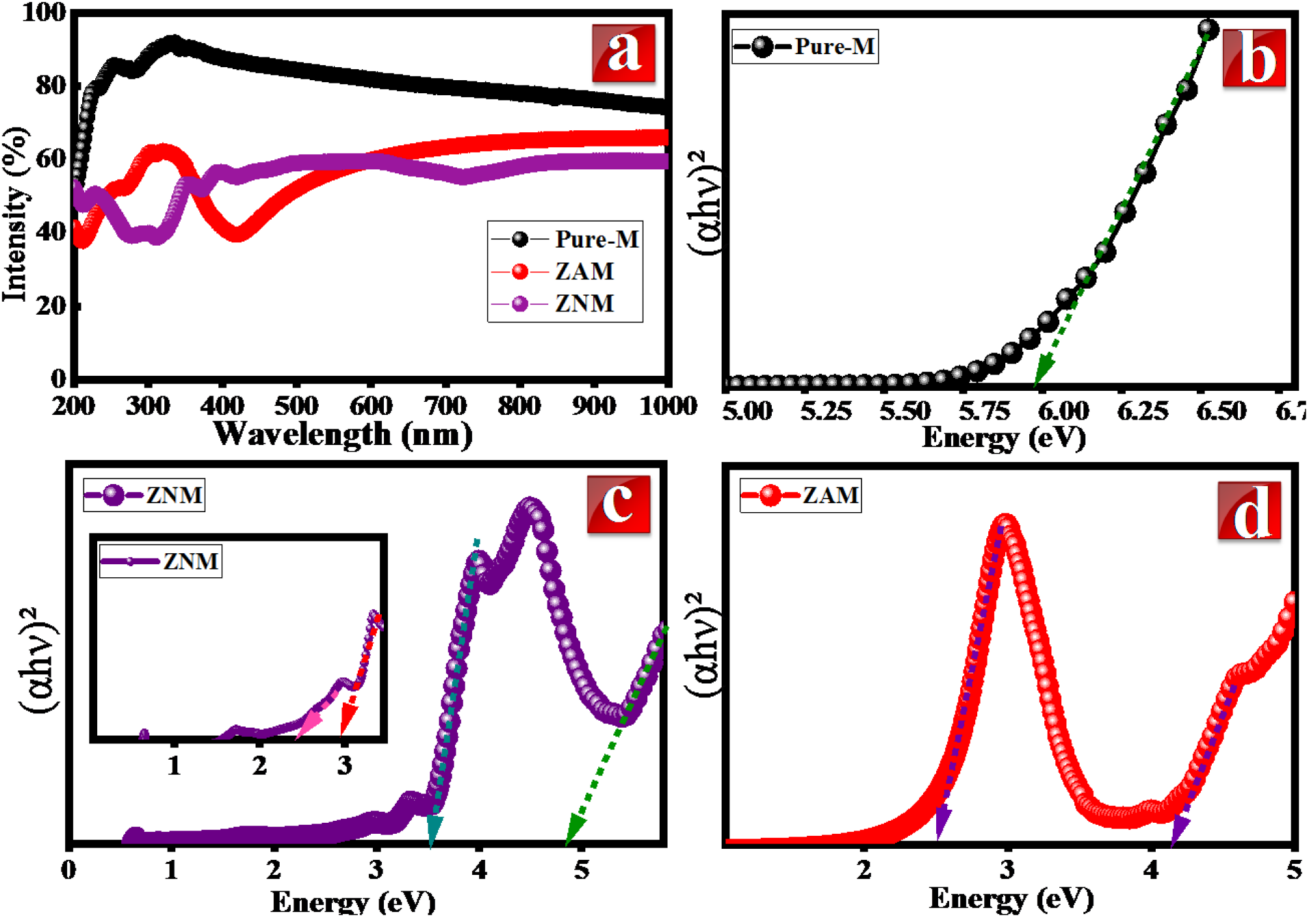
(**a**) Diffuse reflectance spectra and (**b**, **c** and **d**) band gap of MCM-41, ZNM and ZAM samples.

To assess the impact of Zn/Ag and Zn/Ni metal ions on the optical absorption and bandgap energy (BG) of the MCM-41 lattice, UV-Vis DRS (UV-Vis Diffuse Reflectance Spectroscopy) were measured in the 200–1000 nm range for both pure and metal modified samples. These results are graphically represented in Fig. 7. Notably, the absorption characteristics of the MCM-41 underwent significant changes after metal inclusion. The pure M spectrum displayed great reflectance (above 75%) in the 350–1000 nm range. This indicates a highly scattered and reflective material in the visible and near-infrared regions. MCM-41’s ordered structure likely contributes to this by efficiently reflecting incoming light. Small absorption peak at 279 nm; this suggests weak absorption of ultraviolet light, possibly due to electronic transitions within the material. The inherent nature of the pure silica matrix, devoid of π and n electrons, results in limited responsiveness to light radiation.

A small absorption red-shift occurs upon metal ion inclusion, indicative of the construction of –O–M–O–Si–O– linkages, suggesting the establishment of a chemical bond between silicon and metal atoms via oxygen. Consequently, metal species in the M-MCM-41 sample could be exist in tetrahedral coordination. The introduction of dopants like Ni/Zn and Ag/Zn leads to increased light absorption across the spectrum, evident by the lower reflectance values. This suggests the dopant ions introduce new electronic states that interact with light. Compared to pure MCM-41, the doped samples exhibit additional absorption peaks at various wavelengths. These peaks correspond to the electronic transitions within the dopant ions themselves or their interaction with the MCM-41 framework.

For Ni/Zn doping; the presence of additional absorption peaks at 312, 372, 420, and 728 nm likely arises from the d-d electron transitions within the incorporated Ni and Zn ions. The absorbance peak observed at 312 nm and 372 nm are assigned to ZnO nanoparticles absorption^49–51^. The band at about 372 nm links with the BG of ZnO; while the 312 nm peak exhibits a blue shift relative to the bulk ZnO wavelength. A clear correlation exists between decreasing nanoparticle size and a systematic absorption blue shift, attributable to the quantum size effect^52^. In the visible region, absorption bands appear at 420 and 728 nm which are due to the ^3^A_2*g*_ → ^3^T_1*g*_(P)) and 2 ^3^A_2g_ → ^3^T_1g_(F)) transition of Ni^2+^ ions in an octahedral or pseudo-octahedral configuration^53^. On the other hands, the weak bands at 550 nm, 600–645 nm are associated with tetrahedrally coordinated Ni^2+^ species^54^.

Upon the Ag/Zn inclusion; the intense absorption at 209 nm and moderate at 271 nm could be ascribed to Ag-Zn or Ag-Zn-MCM-41 interactions. The observed weak band centered at 312 could be assigned to Ag_n_ clusters^55,56^. The wide absorption band 250 to 450 nm (centered at 416 nm) could be ascribed surface plasmon resonance of the Ag nanoparticles^57,58^. No adsorption bands were observed at 360–380 nm that were corresponding to polymerized ZnO indicating that most of Zn species existed in MCM-41 framework. Moreover, the intensity of peak at 312 nm (observed in ZNM) decreased upon Ag doping, which indicated that the using of Ag/Zn doping favored formation of Ag_n_ clusters and lead to more inclusion of Zn ions in MCM-41 structure.

The E_g_ of pure M, ZAM and ZNM was estimated through Tauc plot analysis. This method involves plotting (αhν)ⁿ against photon energy (hν) and extrapolating the linear region to the x-axis to determine E_g_^59^. To obtain absorption data for Tauc plot analysis, RS were transformed to absorption spectra using the Kubelka-Munk equation. The linear relationship observed between (αhν)^2^ and photon energy for the fundamental transition indicates a direct band gap nature for MCM-41, as depicted in Fig. 7b. The calculated BG energy for pure M is 5.98 eV.

Figure 7(c, d) shows that the DRS spectra of ZAM and ZNM exhibit a red-shifted absorption edge with an extended absorption tail in the vis-light range. This phenomenon is associated with formation new energy states within the SiO_2_ BG due to metal inclusion. A noteworthy BG decline from 5.98 eV for pure M to 4.92 eV in ZNM and 4.25 eV in ZAM is observed.

Furthermore, additional absorption bands emerge in the spectra of ZNM and ZAM samples, corresponding to the bandgaps of ZnO, NiO, and AgO. Specifically, absorption bands at 3.52 and 2.95 eV align closely with a modified ZnO bandgap, while bands at 2.47 and 2.51 eV in ZNM and ZAM approximate the bandgap energies of NiO and AgO, respectively. These results authenticate the heightened ability of modified sample ZAM and ZNM for absorbing a wide range of visible radiation in comparison with pure M.

The detected decline in BG is recognized to oxygen vacancies (Vo) generating as well as inclusion of impurity states in BG of MCM-41 lattice. This is facilitated by the substitution of Si^4+^ ions with divalent Zn^2+^, Ni^2+^, and Ag^+^ ions. The replacement of tetravalent Si^4+^ with divalent ions necessitates (Vo) formation to maintain charge neutrality. Consequently, the introduction of metal ions and oxygen vacancies generates impurity energy levels within the bandgap, leading to a reduction in the optical bandgap^60^. These findings underscore the potential of these tailored materials for solar energy photocatalysis applications.

$${\text{ZnO}}\xrightarrow{{{\text{SiO}}_{2} }}{\text{Zn}}_{{{\text{Si}}}}^{{\prime\prime}} + {\text{V}}_{{{{\ddot{\text{O}}}}}}$$

$${\text{AgO}}\xrightarrow{{{\text{SiO}}_{2} }}{\text{Ag}}_{{{\text{Si}}}}^{\prime\prime\prime} + {\text{V}}_{{{{\ddot{\text{O}}}}}}$$

$${\text{NiO}}\xrightarrow{{{\text{SiO}}_{2} }}{\text{Ni}}_{{{\text{Si}}}}^{\prime\prime} + {\text{V}}_{{{{\ddot{\text{O}}}}}}$$

### Infrared spectra

**Fig. 8:**
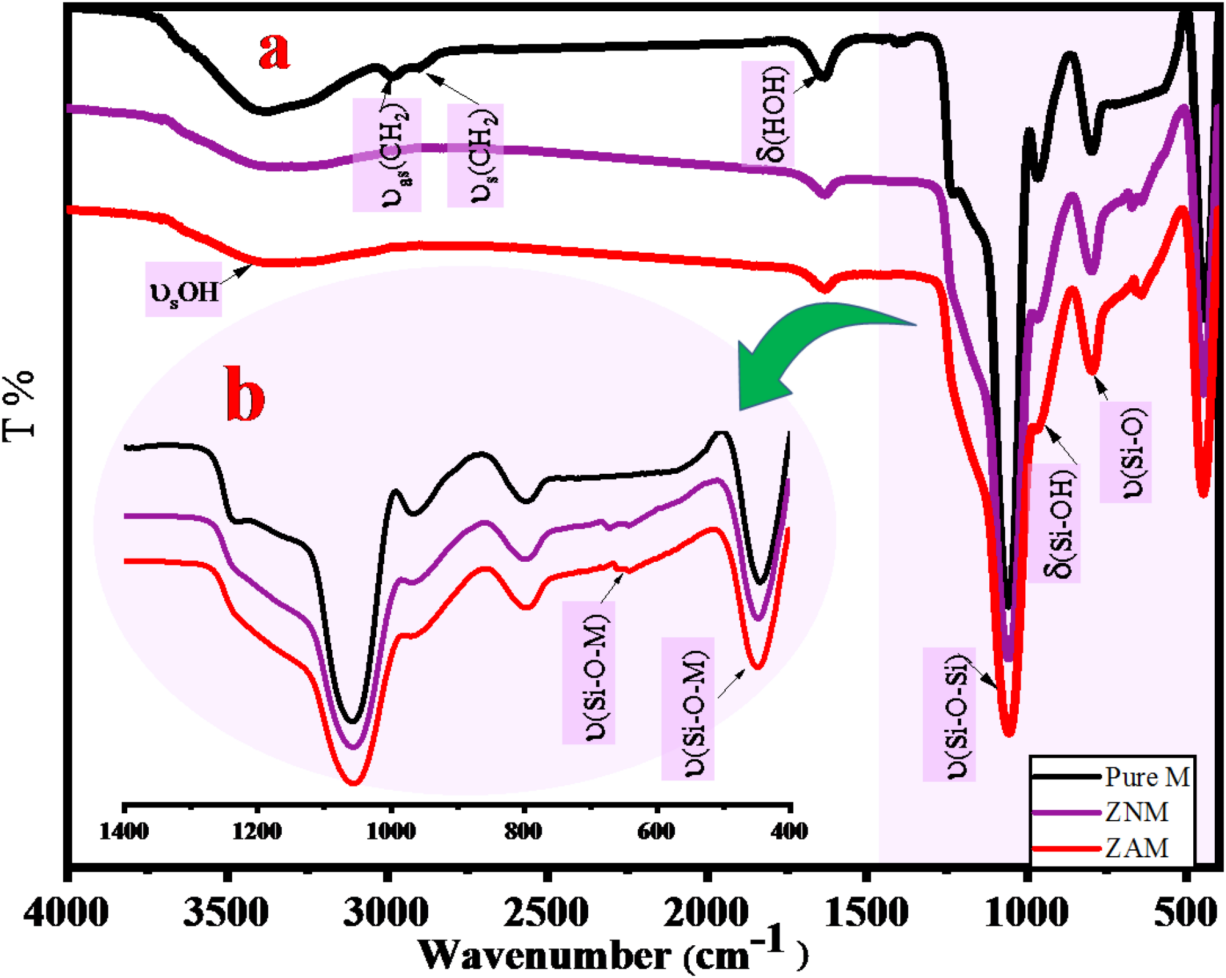
Fourier Transform Infrared (FTIR) spectra of pure M, ZNM, and ZAM samples. (**a**) Full spectrum (400–4000 cm^−1^) and (**b**) Expanded view of the fingerprint region (400–1400 cm^−1^).

The IR spectra of pure M, ZAM and ZNM are presented in Fig. 8a. An expanded view of the 400–1400 cm^− 1^ region is provided in the inset of Fig. 8b. Vibrational bands were observed in the 400–4000 cm^− 1^ range typical to structural framework^61,62^. Within the pure MCM-41 sample, a broad band is detected at 3380 cm^− 1^ attributed to hydrogen-bonded υ(SiOH) group, influenced by H_2_O_ads_^63,64^. Additionally, the 1634 cm^− 1^ peak is credited to the H_2_O_ads_ bending mode. Asymmetric υ Si-O-Si are observed at 1229 and 1062 cm^− 1^, while symmetric Si-OH stretching is indicated by the 963 cm^− 1^ band. While the (Si-O-Si)_sym_ stretching is represented by the 796 cm^− 1^, and the discrete band at 447 cm^− 1^ corresponds to the siloxane υ_bending_ Si-O-Si.

For Zn/Ni and Zn/Ag samples (Fig. 8), the 3380 and 1634 cm^− 1^ bands transmittance intensity declines, with small shifts to lesser wavenumbers. These intensity and shifts variations are indicative of metal ions incorporation in the mesoporous framework, aligning with prior reports^65,66^. More importantly, the bands associated with the υ_sym_ and υ_asy_ of T–O–T (T = Si or M) linkage exhibit some variations, reflecting the inclusion of Zn, Ni, Ag ions in the MCM-41 structure.

Specifically, the υ_stretching_ Si-O-Si at 1062 cm^−1^ in pure M was shifted to 1059.15 cm^−1^ in all modified samples. Additionally, alterations in the intensity and position of the Si-OH stretching band at 963 cm^−1^ shifting to 968 cm^−1^ with Ni incorporation and 970 cm^−1^ with Ag incorporation indicate structural changes in the surface Si-OH groups due to the inclusion of Ni and Ag ions into the Si-MCM-41 structure^67–69^.

Moreover, new bands appeared at 668.20 and 640.18 cm^−1^ following the introduction of Ni and Ag ions into MCM-41, attributed to Si-O-(Ni/Ag) species. Combined evidence from XRD, TEM, SEM, and textural analyses supports the conclusion that metal ions were successfully incorporated into the MCM-41 molecular sieve skeleton, resulting in the formation of M-MCM-41.

### Tetracycline antibiotic removal

**Fig. 9:**
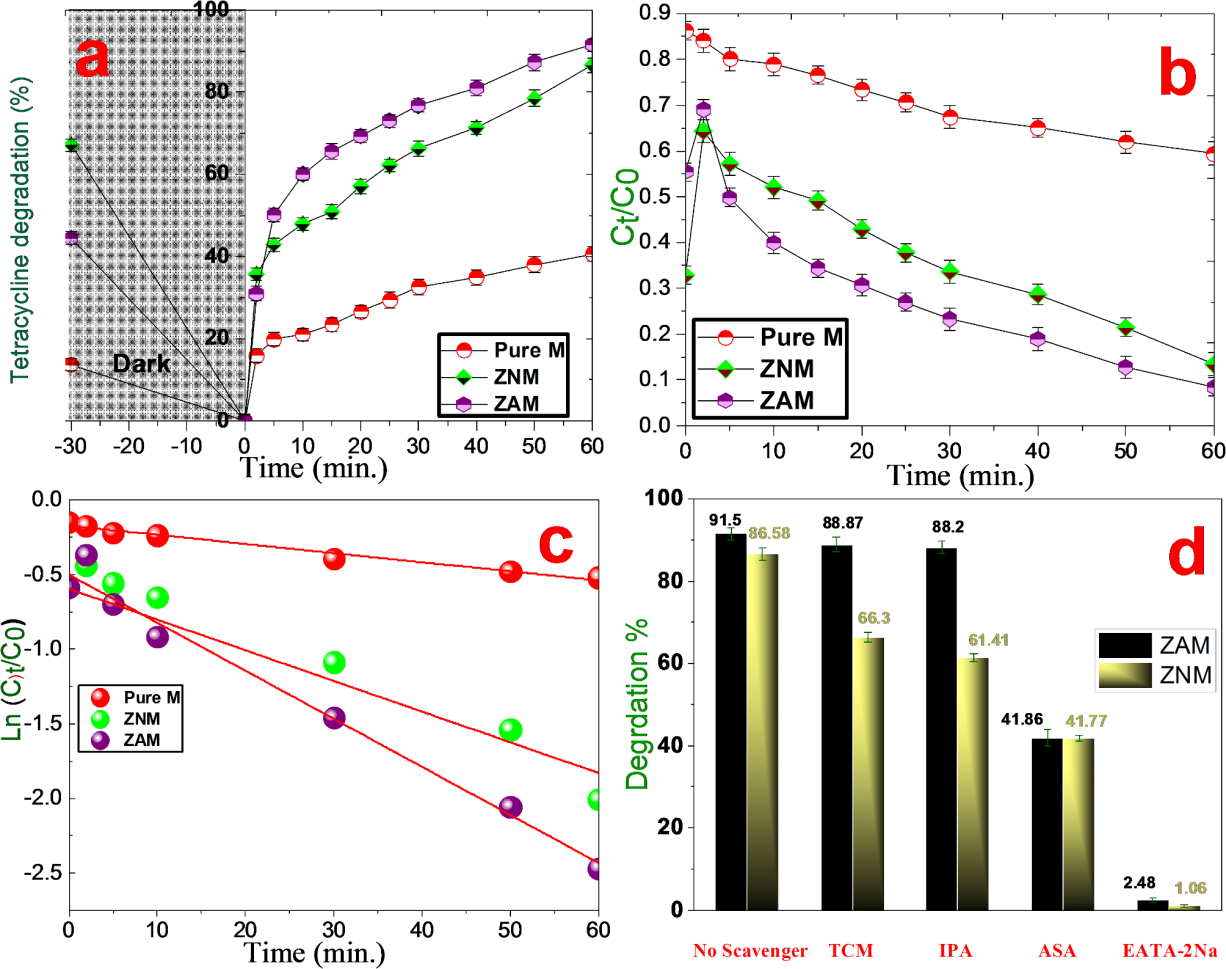
The tetracycline removal efficiency using pure M, ZNM and ZAM samples. (**a**) TC antibiotic degradation efficiency (%) (**b**) the photocatalytic activity (**c**) Kinetic profile, expressed as the natural logarithm of the concentration ratio (C_t_/C_0_) over time, (**d**) the photocatalytic degradation of ZNM and ZAM in the absence and presence of scavengers.

Efficacy of pure M, ZNM, and ZAM samples in removing tetracycline (TC) from aqueous solutions was investigated in Fig. 9a, b. Notably, ZNM and ZAM exhibited significantly higher removal efficiencies (95.56% and 95.30%, respectively) compared to pure M (48.71%) in the same time period. This demonstrates the enhanced removal efficiency of ZNM and ZAM in degrading TC. The results indicated that all samples achieved adsorption equilibrium for TC within 30 min in darkness. The swift removal of TC indicates that adsorption is a primary mechanism in the initial stages of the process. The observed variations in adsorption capacity among the samples can likely be attributed to differences in their pore structures and surface properties. Although pure M boasted the largest surface area (1210 m^2^/g), it surprisingly demonstrated the lowest TC adsorption performance (13.70%), suggesting that surface area alone is not the sole determinant of adsorption efficiency and adsorption of TC molecules on the pure material likely relies on weak van der Waals interactions and H-bonding with mesopore walls.

**Fig. 10:**
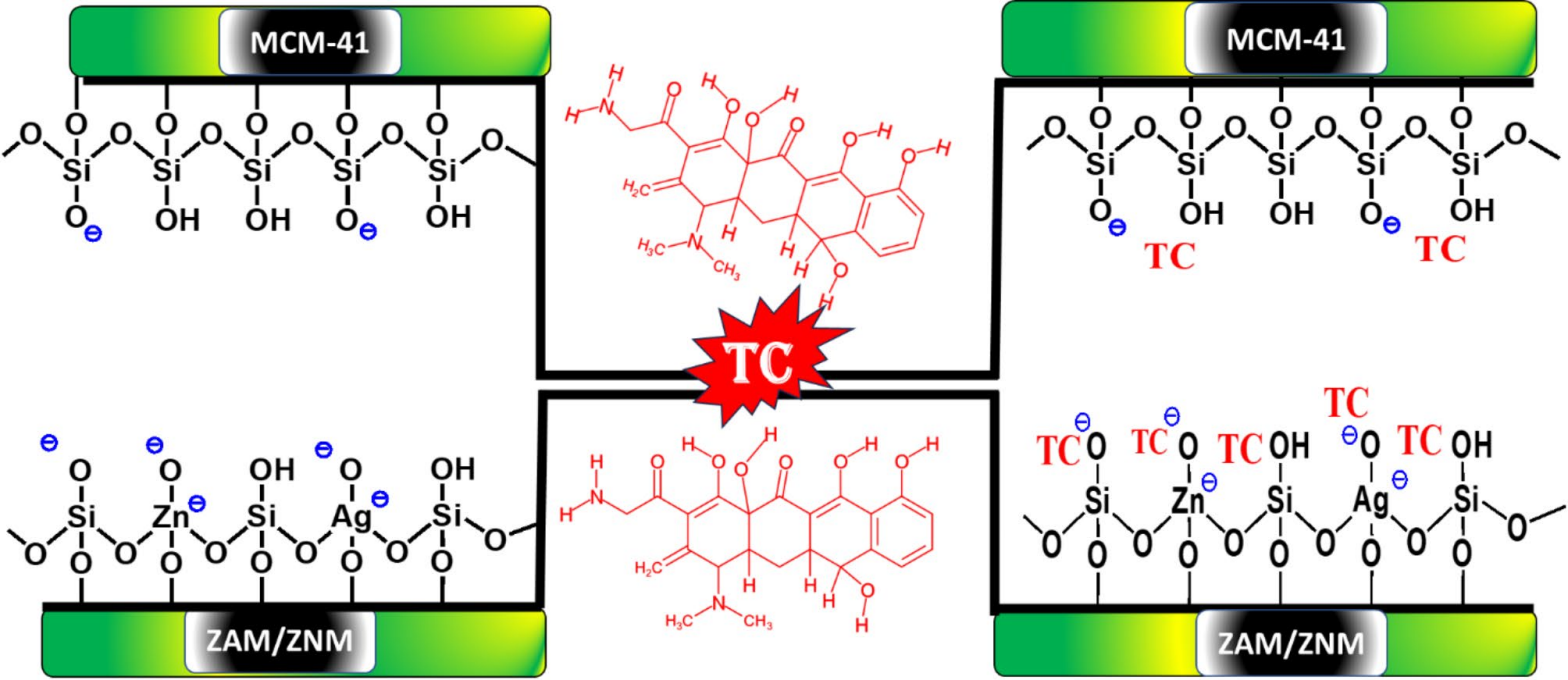
Representation of adsorption of TC on pure M and ZAM, ZNM samples.

In contrast, ZNM and ZAM, incorporating Zn, Ni, and Ag ions, exhibited significantly improved properties by possible enhanced ion-exchange capacity, a higher density of acidic sites, and increased catalytic activity^70^. These characteristics could facilitate stronger electrostatic interactions with TC molecules. The creation of more acidic sites upon MCM-41 framework doping facilitates stronger electrostatic interactions with TC molecules (Fig. 10) and as evidenced by the observed enhancement in TC adsorption capacity for ZNM and ZAM compared to pure MCM-41. The increased negative surface charge likely facilitated stronger electrostatic interactions between the modified materials and the positively charged TC molecules, leading to improved adsorption efficiency. This observed trend is supported by literature reports. Barr and Lishka showed an the excess negative charge on the oxygen atoms and an increase in the covalent character of Si-O bonds in Si/Al binary oxide, which normally leads to a large number of Brönsted acid sites to balance the excess negative charge on the oxygen atoms^71^. Gilles et al. reported that decreasing the Si/Al ratio leads to an increase in the negative charge on oxygen atoms within the material, and results in a corresponding decrease in activation energy. This phenomenon likely arises from the increased electron density on oxygen atoms, which can weaken bonds within the material, facilitating reactant interaction and potentially stabilizing the reaction’s transition state^72^. Dhal et al. reported that Fe-MCM-41 exhibited a higher adsorption capacity for methylene blue compared to pristine MCM-41. This enhancement is likely attributed to the presence of a greater number of negatively charged sites on the surface of Fe-MCM-41, including both negatively charged oxygen atoms (O^−^) and negatively charged iron species (Fe^−^). The increased number of negative sites on the Fe-MCM-41 surface facilitates stronger electrostatic interactions with the positively charged methylene blue dye molecules, leading to enhanced adsorption^73^.

This electrostatic attraction, rather than surface area, was the primary driving force for TC adsorption in ZNM and ZAM^74^. While both ZAM and ZNM materials relied on electrostatic attraction, ZNM’s slightly larger surface area (722.86 m^2^/g) compared to ZAM (700.36 m^2^/g) resulted in a higher adsorption capacity (67.197%) compared to ZAM (44.56%), demonstrating that surface area remains a factor up to a certain point.

After the initial dark adsorption phase, photocatalysis became the primary mechanism for tetracycline (TC) removal. Under solar irradiation, the photodegradation efficiency of TC reached 40.57% for pure M within one hour. In contrast, ZNM and ZAM exhibited significantly higher photocatalytic efficiencies, reaching 86.575% and 91.53%, respectively. This remarkable difference highlights the superior photocatalytic performance of ZNM and ZAM compared to pure M. Notably, ZAM demonstrated the highest degradation efficiency for the TC antibiotic. The enhanced photocatalytic activity of ZNM and ZAM can likely be attributed to the presence of metal species, which may influence their light absorption properties. Compared to existing literature (Table 2), the ZNM and ZAM are considered promising photocatalysts materials for tetracycline (TC) degradation. Their superior performance can be attributed to several factors, including a tailored bandgap and enhanced charge carrier generation. The introduction of metal species, as confirmed from the optical study results, has narrowed the bandgap (5.98 eV for pure M to 4.92 eV in ZNM and 4.25 eV in ZAM) and extended the absorption bands into the visible region (2.47 and 2.51 eV in ZNM and ZAM, respectively), enabling the absorption of a wider spectrum of light.

**Table 2:** Photocatalytic TC degradation using various photocatalysts.

Catalyst	TC initial conc. (mg/L)	Time (min.)	Catalyst dosage (g/L)	D (%)	Refs.
MOF/CCAC	50	120	1	98	^75^
In_2_O_3_/Co_3_O_4_	50	120	2	80	^76^
Carbon dot decorated NiFe_2_O_4_/g-C N	20	120	0.5	93	^77^
ZnO-RSA	50	30	0.7	79.76	^78^
ZnO-Ni-RSA	50	30	0.7	92.68	^78^
AgBr/CeO_2_	30	120	0.05	77.80	^79^
CeO_2_NRs	20	90	0.02	89.36	^33^
SrO-mpg-CN/TiO_2_	10	180	-	91.73	^80^
CDs/g-C_3_N_4_/MoO	20	90	-	88.4	^81^
Pure M	40	60	1	48.71	Present study
ZNM	40	60	1	95.596	Present study
ZAM	40	60	1	95.30	Present study

These modifications arise from the introduction of new energy levels within the SiO_2_ bandgap. The reduced bandgap, likely caused by the incorporation of metal species, allows for the absorption of a wider range of light wavelengths^23^. This leads to the generation of a higher density of charge carriers, which is crucial for driving the photocatalytic degradation process. The presence of energy states levels related to zinc, nickel, and silver ions in the MCM-41 structure enhanced photocatalytic activity by capturing electrons and extending their lifetime, reducing electron-hole recombination through the formation of Schottky barriers or interfacial heterojunctions. This allows for the more production of reactive oxygen species (ROS) that degrade TC molecules^29^. From a different perspective, presence of Zn, Ni and Ag can effectively facilitate electron transfer through the conduction band (CB) of MCM-41, reducing electron-hole recombination and improving photocatalytic conversion efficiency^32^. Thus, the enhanced photocatalytic activity of ZNM and ZAM can be attributed to both improved photon absorption and charge separation. UV-Vis DRS analysis revealed reduced bandgap energies for ZNM and ZAM, enabling them to absorb a wider range of visible light and generate more photoexcited charge carriers. Furthermore, photoluminescence (PL) spectroscopy indicated a decrease in electron-hole recombination in ZNM and ZAM compared to pure MCM-41, suggesting more efficient charge separation. This combination of increased photon absorption and reduced charge recombination contributes to the superior photocatalytic performance of these bimetallic catalysts. Moreover, the improved quantum efficiency of ZNM and ZAM is also attributed to the beneficial electron transport properties of the incorporated ions. The mesoporous structure of ZNM and ZAM provide an extra advantage for enhancing accessibility TC molecules to active sites, facilitating reactant and product diffusion throughout the photocatalyst. This allows for photocatalytic reactions to occur both on the surface and within the porous structure, potentially leading to a higher degradation rate.

Figure 9c illustrates the logarithmic decrease in TC concentration (ln(C_t_/C_0_)) over time for the pure M, ZNM, and ZAM catalysts to degrade tetracycline (TC) under solar light was estimated. As presented in Table 3, The high correlation coefficients obtained specify a good agreement between the experimental data and the pseudo-first-order model, confirming its suitability for describing the TC degradation by these photocatalysts. The rate constant (k_1_) for this degradation process was determined by linear regression analysis, as shown in the following equation: ln(C_t_/C_0_) = -k_1_t^82^. The rate constant k_1_ (min^− 1^) for pure M, ZNM and ZAM catalysts were calculated. Among the catalysts tested, ZAM exhibited the highest (k_1_) of 0.03298 min^− 1^, indicating that inclusion of silver (Ag) in the Zn/MCM-41 composite significantly boosted photocatalytic activity. This improvement could be credited to the creation of Ag/Zn/MCM-41 heterojunction structure, which promotes synergistic effects as evidenced by the superior performance of the ZAM photocatalyst^32^.

**Table 3:** Kinetic parameters for TC drug removal using pure M, ZNM and ZAM samples.

Samples	Pseudo-first order		Pseudo-second order	
	K_1_ (min^− 1^)	*R* ^2^	K_2_ (min^− 1^)	*R* ^2^
Pure M	-0.0059	0.9811	2.43944E-4	0.96911
ZNM	-0.02507	0.9849	0.00678	0.90289
ZAM	-0.03298	0.98535	0.00723	0.94081

### Effect of scavengers

During the photodegradation of tetracycline (TC), the highest degradation efficiency was observed in the absence of any scavengers: 91.53% over the ZAM catalyst and 86.575% over the ZNM catalyst after 60 min of solar light irradiation. This high degradation rate is likely because all reactive oxygen species (ROS) produced during light exposure were fully involved in the reaction, with no external interference. These results serve as a baseline for understanding the overall efficiency of the catalysts under ideal conditions. When various scavengers were introduced to the system, the degradation efficiency over the ZAM catalyst showed a marked decrease, which provides crucial insight into the role of different ROS in the photodegradation process. In the presence of TCM, a scavenger for electrons, the efficiency only slightly dropped from 91.53 to 88.87%, suggesting that electrons are present but contribute minimally to the degradation process. This minor drop shows that while electrons participate, they are not the dominant species driving the reaction. A similar trend was observed when IPA (a scavenger for hydroxyl radicals) was added, reducing the degradation efficiency to 88.20%. This slight reduction implies that hydroxyl radicals play a minor role in the photodegradation of TC. The relatively small impact of both TCM and IPA suggests that electrons and hydroxyl radicals are not the primary contributors to the high degradation efficiency seen without scavengers. In clear contrast, the introduction of ASA (a scavenger for superoxides) led to a more substantial drop in efficiency, down to 41.86%. This indicates that superoxide radicals play a significant role in the degradation process. The nearly 50% reduction highlights the importance of superoxides as active species in breaking down TC molecules. The most dramatic decrease in efficiency was observed with EDTA-2Na, a scavenger for holes, where the degradation efficiency plummeted to just 2.48%. This sharp decline indicates that holes are the most critical ROS in this system, driving the bulk of the degradation process. The near-complete suppression of TC degradation in the presence of EDTA-2Na demonstrates that without holes, the reaction is almost entirely inhibited^32,80^.

A similar pattern was noted with the ZNM catalyst. When no scavengers were used, the degradation efficiency was 86.575%. However, the addition of EDTA-2Na reduced this drastically to only 1.06% Fig. 9d, reinforcing the conclusion that holes are the dominant species responsible for the catalytic reaction. Meanwhile, TCM only slightly lowered the efficiency to 66.30%, again confirming that while electrons are involved, they do not play a central role in the overall degradation mechanism.

The progressive decrease in degradation efficiency observed with the use of different scavengers offers valuable insights into the relative importance of various ROS in the photodegradation of TC. Photogenerated holes clearly emerge as the most significant species, followed by superoxides, while electrons and hydroxyl radicals appear to play a more secondary role. These findings not only clarify the underlying mechanism of the reaction but also emphasize the importance of targeting holes and superoxides when optimizing photocatalysts for enhanced degradation efficiency.

#### Zero point of Charge and effect of pH

**Fig. 11:**
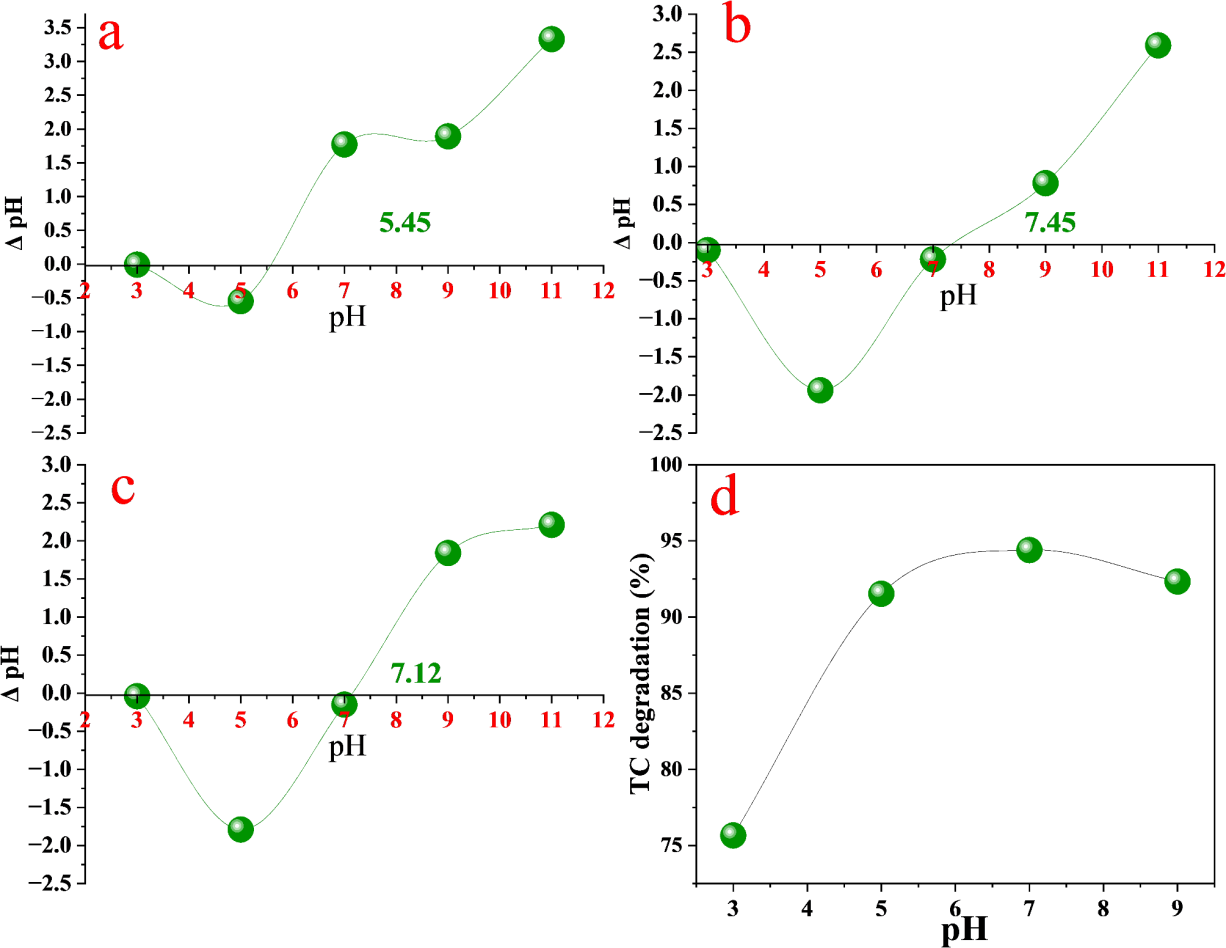
Zero-point charge (pH_ZPC_) of (**a**) pure M, (**b**) ZNM, (**c**) ZAM, (**d**) The tetracycline removal efficiency of ZAM at different pH values.

Figure 11a–c illustrates the impact of Zn/Ag and Zn/Ni doping on the point of zero charge (PZC) of MCM-41. Doping increased the PZC value from 4.45 in pure MCM-41 to 7.48 and 7.12 for ZNM and ZAM samples, respectively. A higher PZC value is theoretically linked to improved adsorption performance. The pH of the solution plays a crucial role in influencing the surface charge of the ZAM photocatalyst and tetracycline (TC) molecules, thereby affecting TC adsorption onto the ZAM surface and influencing photocatalytic degradation efficiency. As depicted in Fig. 11d, the photodegradation efficiency of TC using ZAM exhibits strong pH dependence. This behavior is attributed to the amphoteric nature of TC, which causes its charge to vary with solution pH. TC degradation efficiency increased between pH 3 and 5, peaked at pH 7, and declined from pH 7 to 9. This trend is governed by electrostatic interactions between the pH-sensitive surface charge of ZAM and the changing speciation of TC molecules.

**Fig. 12:**
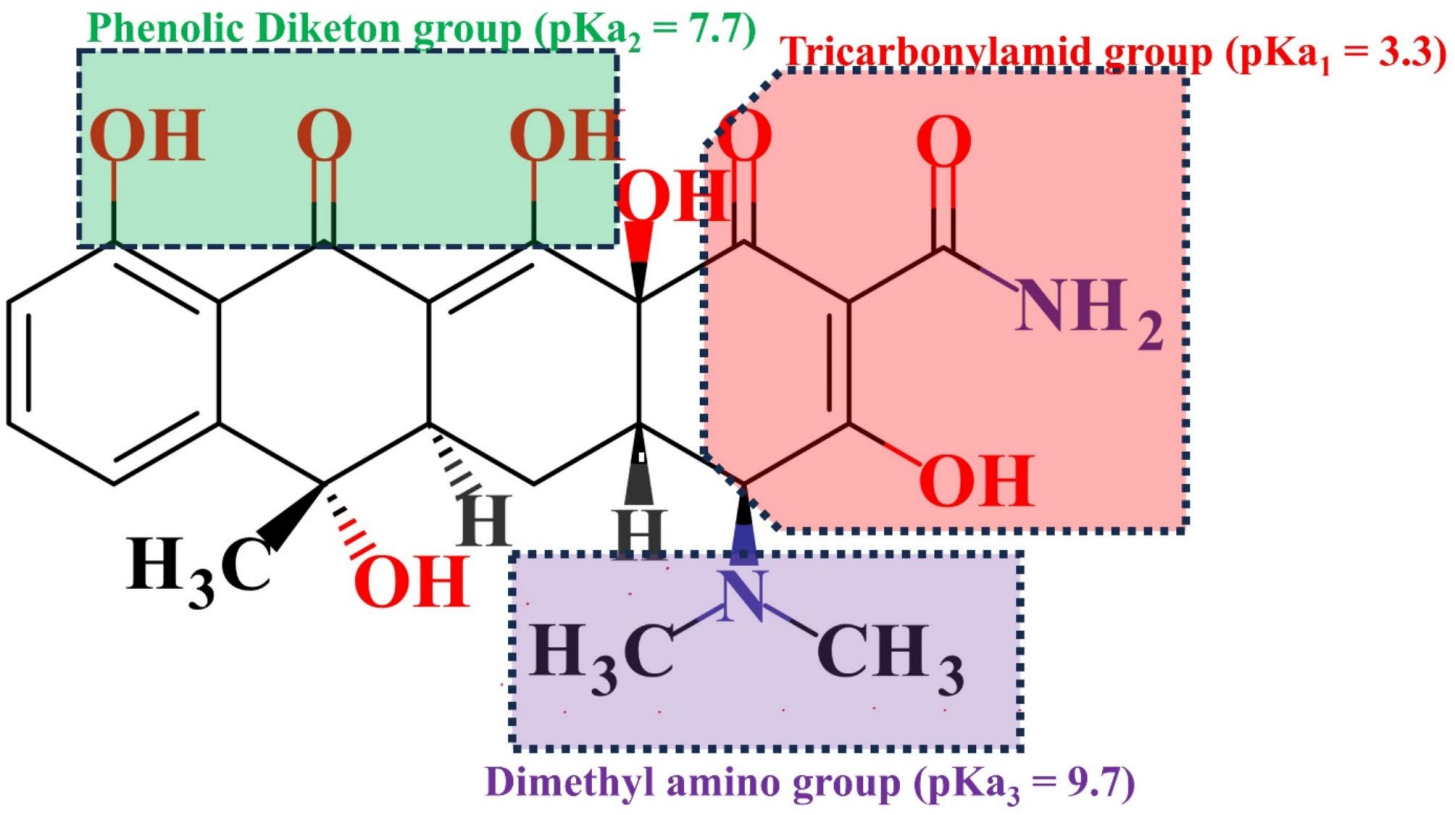
Representation of tetracycline structure with its multiple ionizable functional groups.

TC contains multiple ionizable functional groups, causing its speciation to shift with pH (Fig. 12). TC exists as a cation (H_2_TC^+^) in acidic conditions, transitions to a zwitterionic form (H_2_TC^0^) between pH 3.3 and 7.7, and becomes predominantly anionic (HTC^−^/TC^2−^) at higher pH values^78^. Within the pH range of 5–7, TC primarily adopts a zwitterionic form, while the ZAM surface remains slightly positively charged (pH_zpc_ = 7.12, Fig. 11a).

Although TC is overall neutral in this range, electrostatic interactions occur between its negatively charged tricarbonylamide groups and positively charged ZAM sites, leading to maximum adsorption. At pH values below 5, electrostatic repulsion between the cationic TC species and the positively charged ZAM surface impedes adsorption. Above pH 7.12 (ZAM’s PZC), the negatively charged ZAM surface repels anionic TC species, resulting in reducing adsorption and consequently total photodegradation. This dynamic interplay of electrostatic forces accounts for the pH-dependent variation in TC degradation, with optimal degradation observed near the ZAM’s PZC.

### Possible degradation mechanism

The results suggest that photocatalytic oxidation of tetracycline (TC) by the synthesized materials likely follows a three-step mechanism^83,84^. The first step involves the TC molecules ads. on the photocatalyst (pure M, ZNM, or ZAM) surface. This adsorption process brings the TC molecules into close contact with catalyst active sites, thereby facilitating their subsequent degradation. In the second step, light irradiation occurs. The photocatalyst material (e.g., the metal oxides in ZNM and ZAM) absorbs photons with sufficient energy (typically visible light). This absorption excites electrons within the catalyst, promoting them from ground state to higher energy level. The final step involves a series of charge transfer processes and the generation of reactive species. Thus, upon sunlight irradiation, the photocatalysts (pure M, ZNM, and ZAM) initiate a series of processes that lead to the degradation of tetracycline (TC).

Understanding the photocatalytic properties requires knowledge of its electronic band structure, specifically the energy levels of the valence band (VB) and conduction band (CB). These energy levels dictate the material’s ability to absorb light, generate electron-hole pairs, and participate in redox reactions crucial for pollutant degradation. The valence band (VB) and conduction band (CB) energy levels of a semiconductor can be theoretically determined using the following equations^85–87^: E_VB_ = X - E_e_ + 0.5E_g_; E_CB_ = E_VB_ – E_g_ where: E_VB_: Energy of the top of the valence band, E_CB_: Energy of the bottom of the conduction band, X: Geometric mean of the Mulliken electronegativities of the constituent atoms in the semiconductor, Ee: Energy of free electrons on the hydrogen scale (a constant value of 4.5 eV vs. NHE), Eg: Bandgap energy of the semiconductor. These equations were employed to calculate the VB energy levels of pure ZAM, ZNM, ZnO, AgO, and NiO, with the results summarized in Table 4.

**Table 4:** Band gap, conduction band, valence bands energy values of pure M, ZNM and ZAM samples.

Sample	BG	CB	VB
Pure M	5.98	-1.020	4.959
ZAM	4.25	-0.16	4.08
ZNM	4.92	-0.499	4.420
NiO	2.47	0.096	2.566
ZnO	2.95	-0.218	2.731
AgO	2.51	0.029	2.539

**Fig. 13:**
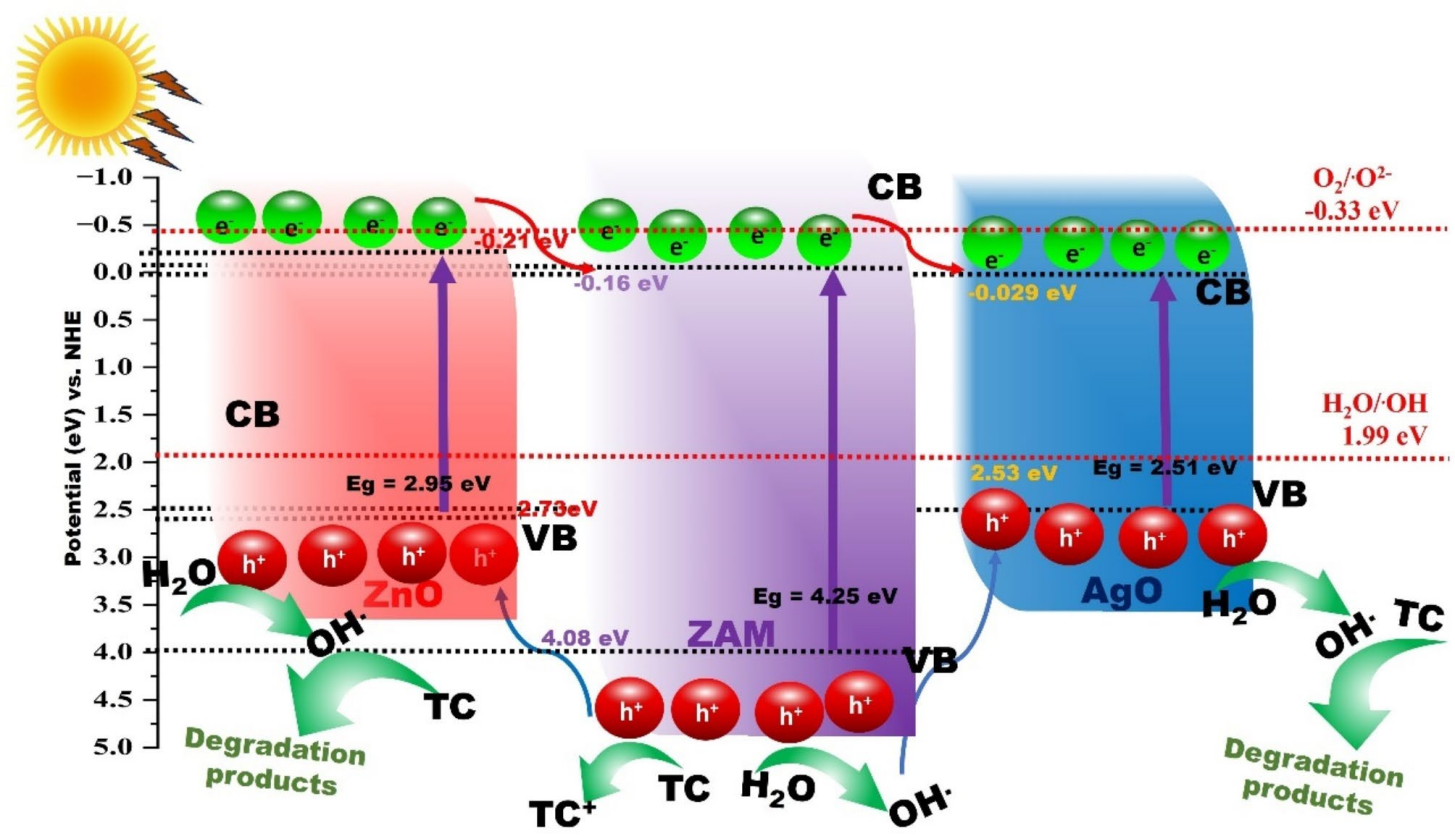
The predicted mechanism for the degradation of tetracycline (TC) over the ZAM.

Based on the experimental results, a proposed photocatalytic mechanism for TC dehydrogenation by the ZAM catalyst is illustrated in Fig. 13. Under light illumination, electrons in the valence bands (VBs) of ZAM, ZnO, and AgO are excited to their respective conduction bands (CBs), concurrently generating an equal number of holes (h^+^) in their VBs. Given that the CB of ZnO (-0.23 eV) is more negative than the CBs of ZAM and AgO (-0.16 eV and 0.029 eV, respectively), photoexcited electrons from the ZnO CB can readily migrate to the CBs of ZAM and AgO. Similarly, since the VB of ZAM (4.08 eV) is higher than the VBs of ZnO and AgO (2.73 eV and 2.53 eV), photogenerated holes in the ZAM VB can transfer to the VBs of ZnO and AgO, enabling the oxidation of TC. The potential of O_2_/^·^O_2_^−^ (-0.33 eV)^88^ is more negative than the CB potentials of ZnO, ZAM, and AgO, preventing the direct reduction of O_2_ to ^·^O_2_^−^ radicals by electrons in the CBs of these semiconductors. Conversely, the potential of ^·^OH/H_2_O (+ 1.99 eV)^88^is lower than the VB potentials of all participating species (ZAM: 4.45 eV, ZnO: 2.73 eV, AgO: 2.53 eV). Consequently, holes in the VBs of ZAM, ZnO, and AgO can oxidize H_2_O to produce ·OH radicals. The facilitated transfer of electrons from the ZnO CB to the CBs of ZAM and AgO, coupled with the transfer of holes from the ZAM VB to the VBs of ZnO and AgO, effectively promotes the separation of electron-hole pairs, thereby enhancing the photocatalytic activity of the ZAM catalyst. Based on the scavenger experiments, it is clear that the superoxide radicals holes (h^+^) are the most active reactive oxygen species (ROS) in the photodegradation process. While hydroxyl radicals (OH•) and electrons (e^−^) contribute to the reaction, their role is less significant. The holes are responsible for attacking and breaking down the TC molecules into smaller, less harmful compounds. Furthermore, the heterojunctions formed between zinc, nickel, silver, and the MCM-41 support play a crucial role in enhancing photocatalytic performance. These heterojunctions promote the separation and extended lifetime of the charge carriers by forming barriers or interfacial junctions, which help capture electrons and prevent their recombination with holes. This extended charge separation is key in reducing electron-hole recombination and in improving the efficiency of TC degradation under solar light^29^. The predicted mechanism involved in the degradation of tetracycline (TC) over the synthesized catalyst (ZAM) could be illustrated in the following (Fig. 13).

### Photoluminescence analysis (PL)

**Fig. 14:**
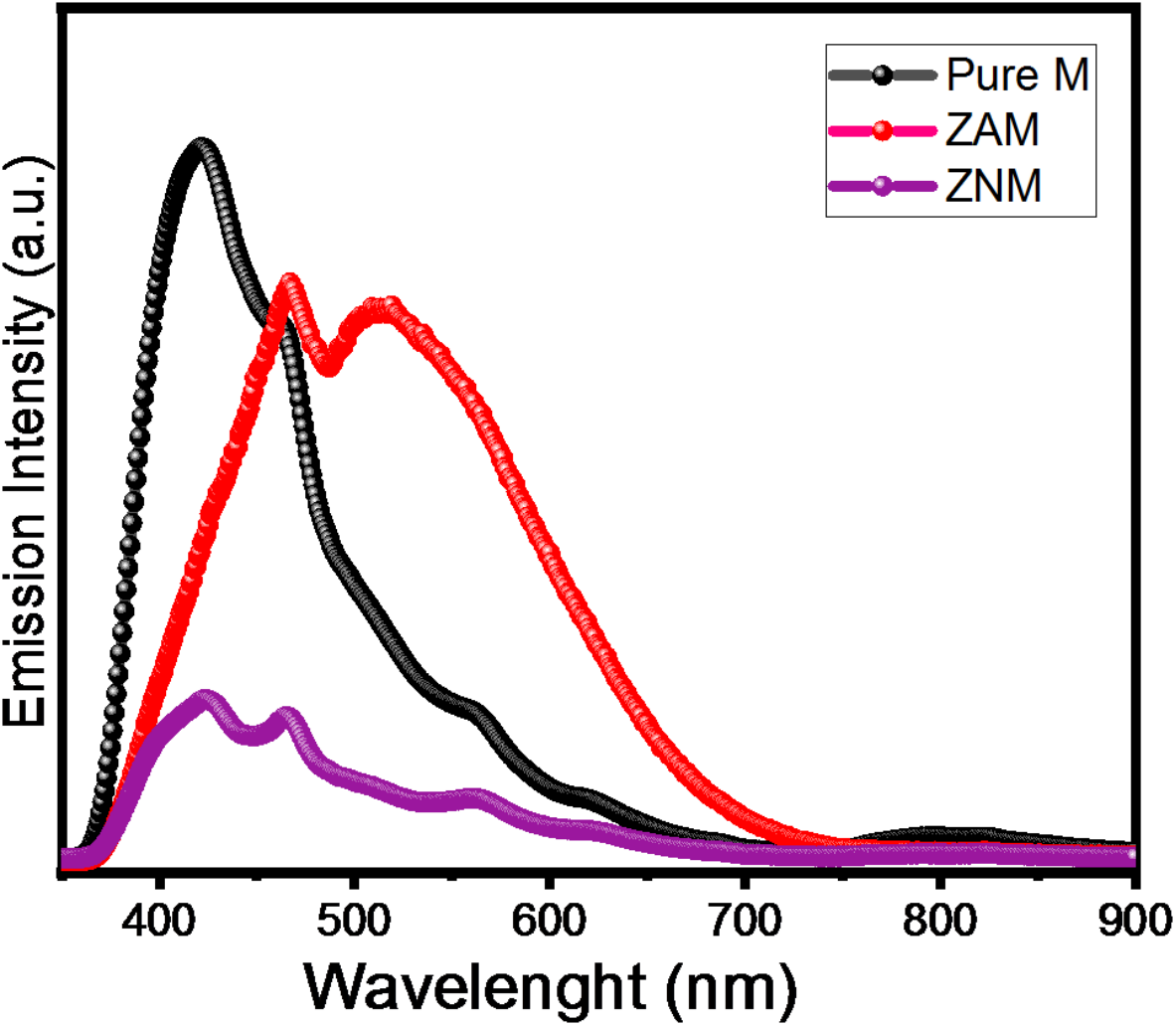
PL spectra of pure M, ZNM and ZAM samples. The excitation wavelength is 325 nm.

PL was conducted to explore the electronic character of the materials and how it cornubites to photocatalytic activity. The PL spectra were acquired utilizing an λ_excitation_ of 325 nm and presented in Fig. 14. The pure M spectrum exhibits two prominent blue emission bands centered at approximately 422 nm (2.94 eV) and 467 nm (2.65 eV). These emission peaks are commonly associated with the recombination of photogenerated charge carriers and Vo within oxygen-deficient silica^89,90^. Alike emission at 3.1 eV in silica was reported by Kohketsu et al., suggesting that silicon atoms perform as luminescence sites^91^. Additionally, 2.76 eV band is associated with energy transfer via the triplet-to-ground state neutral Vo transitions^92,93^.

Interestingly, the blue emission undergoes redshifts with increasing oxygen deficiency in ZAM and ZNM samples. ZAM displays emission peaks at 467 and 516 nm, while ZNM exhibits peaks at 425 and 465 nm. This correlation between blue emission peak position and oxygen deficiency aligns with previous studies on oxygen-deficient silica.

Furthermore, the PL intensity diminishes with both Zn/Ag and Zn/Ni doping, but the reduction is more pronounced in Zn/Ni-doped samples. This decrease in PL intensity suggests a reduction in electron-hole recombination probability upon metal ion doping, leading to a diminished green emission in nickel-doped samples compared to pure ZnO.

## Conclusion

This investigation involved the synthesis of pristine and bimetallic (Zn^2+^/Ag^+^, Zn^2+^/Ni^2+^) ions incorporating MCM-41 materials via a surfactant-assisted precipitation method. Comprehensive characterization confirmed the successful integration of metal ions into the MCM-41 framework, leading to alterations in textural and optical properties. XRD analysis confirmed metal incorporation into the MCM-41 framework, while N_2_ adsorption-desorption isotherms indicated a decrease in specific surface area (1210 in pure MCM-41 to 722.86 and 700.36 m^2^/g for ZNM and ZAM, respectively) due to partial pore filling. Notably, the incorporation of metals resulted in a decreased bandgap, enhancing the material’s potential for visible light photocatalysis. The photocatalytic efficacy of the synthesized materials was evaluated by their ability to degrade tetracycline (TC) under solar light irradiation. Both ZNM and ZAM exhibited substantially superior TC removal efficiencies (95.596% and 95.30%, respectively) compared to pure MCM-41 (48.71%). Furthermore, under irradiation, ZNM and ZAM demonstrated significantly higher photodegradation efficiencies for TC, achieving 86.575% and 91.53%, respectively, compared to 40.57% for pure M. These findings emphasize the enhanced performance of ZNM and ZAM in TC degradation. The photocatalytic degradation mechanism of TC over ZNM and ZAM revealed that the photogenerated holes clearly emerge as the most significant species, followed by superoxide, while electrons and hydroxyl radicals appear to play a secondary role. Also, the effect of pH on TC photodegradation using ZAM sample was evaluated and it was found that the TC degradation was enhanced by pH increment till pH 7 (94.40%), and then it decreased at pH 9 (92.33%). The exceptional photocatalytic activity of ZNM and ZAM can be attributed to factors such as tailored bandgap, efficient charge carrier generation, and the synergistic effects of zinc, nickel, and silver ions within the MCM-41 matrix. These results collectively highlight the potential of ZNM and ZAM as promising visible photocatalysts for the remediation of antibiotic-contaminated water bodies.

## Data Availability

Data will be made available on request; Contact person: Mohammed Ahmed Wahba; mohamedwahba12@gmail.com.
